# Pd‐Catalyzed Cross‐Coupling of Alkylbisboronic Esters

**DOI:** 10.1002/open.202500195

**Published:** 2025-04-10

**Authors:** Philip P. Scott, Christopher E. Baird, Elizabeth A. Kraichely, Liam M. Radeke, Timothy J. Barker

**Affiliations:** ^1^ Department of Chemistry and Biochemistry College of Charleston 66 George Street Charleston SC 29424 USA

**Keywords:** alkylboronic esters, borons, palladium, Suzuki

## Abstract

A Pd‐catalyzed cross‐coupling between alkylbisboronic esters and aryl bromides is described. A variety of aryl bromides are found to be competent substrates in the reaction. Different alkyl chain lengths of the alkylbisboronic esters are examined, and a two‐carbon linker is found to be of the optimal chain length. Several competition experiments are performed to better understand the mechanism of the reaction.

## Graphical abstract

A reaction that selectively couples only one of two identical boronic esters in an alkylbisboronic ester is described. Investigations into the chain length of the alkylbisboronic ester found that the 2‐carbon linker is the most effective substrate under the reaction conditions.

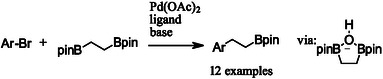

## 1 Introduction

The Suzuki–Miyaura reaction has found broad applications in organic synthesis.^[^ ^1^ ^]^ The synthesis of biaryl compounds is primarily achieved through the Suzuki–Miyaura reaction between an aryl halide and an arylboron compound.^[^ ^2^ ^]^ Recently, the use of alkylboron reagents in Suzuki–Miyaura reactions has also become a common approach.^[^ ^3^ ^]^ Due to the simplicity of this reaction, iterative couplings involving alkylboron reagents have proven to be highly effective.^[^ ^4^, ^5^ ^]^ These strategies often employ different protective groups on the boron to control the rates of transmetalation.^[^ ^6^ ^]^ The removal of these protective groups enables the sequential coupling of the second C—B bond. Additionally, methods have been developed to selectively react one boronic ester in the presence of another similar boronic ester, typically leveraging differences in electronic or steric properties of the C—B bonds to facilitate selective coupling.^[^ ^7^, ^8^, ^9^, ^10^, ^11^, ^12^, ^13^ ^]^ Our research group was interested in examining this cross‐coupling using alkylbisboronic ester substrates, where the boronic esters were in electronically and sterically identical environments. Related Palladium‐catalyzed desymmetrization reactions of 1,2‐diborylcycloalkanes have recently been reported.^[^ ^10^, ^14^, ^15^ ^]^

The rationale for mono‐coupling selectivity in this reaction is based on the previously reported rate acceleration in transmetalation to Pd from a 1,2‐disubstituted bisboronic ester.^[^ ^6^, ^13^ ^]^ Prior ^11^B nuclear magnetic resonance spectroscopy (NMR) and computational studies are consistent with an oxygen‐bridged bisboronic ester intermediate that facilitates transmetalation.^[^ ^10^, ^14^ ^]^ Initial studies with Pd(dppf)Cl_2_ (dppf = 1,1′‐bis(diphenylphosphino)ferrocene) as a catalyst^[^ ^16^ ^]^ with the alklylbisboronic ester **1** and 4‐bromotoluene found there to be excellent selectivity for the monocoupled product (>50:1 as determined by gas chromatography mass spectrometry and NMR).


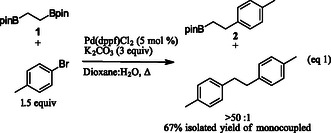


## 2 Results and Discussion

Attempts to expand this method to other aryl bromides under these conditions resulted in reduced yields, prompting a ligand screen to be performed using 4‐bromotrifluorotoluene as a model substrate (**Table** **1**). Ruphos and related ligands, such as SPhos and XPhos, were effective ligands for the aryl bromide substrate examined in this screen.^[^ ^8^, ^14^, ^17^, ^18^ ^]^ The three successful ligands in this ligand screen notably have 2′,6′‐disubstitution on the biphenyl ring of the ligand, making that an important feature for successful cross‐coupling reaction in this system. Substitution at these positions has been reported to block undesired palladacycle formation at those positions that deactivates the catalyst.^[^ ^17^ ^]^ Additionally, it is reported that these groups increase the size and cone angle of the ligand, promoting the monoligated Pd to be the favored species in solution.^[^ ^19^ ^]^

**Table 1:** Ligand screen.

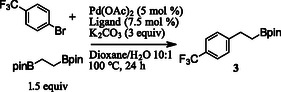		
Entry	Ligand	Yielda)
1	RuPhos	65 (60)b)
2	SPhos	67
3	XPhos	60
4	JohnPhos	27
5	CyJohnPhos	13
6	DavePhos	20
7	Dppf	13
8	D(t‐Bu)pf	38

a) Yield by Internal Standard.

b) Isolated yield.


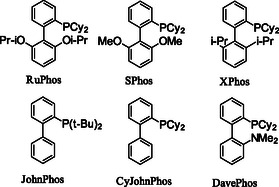


**Scheme 1:**
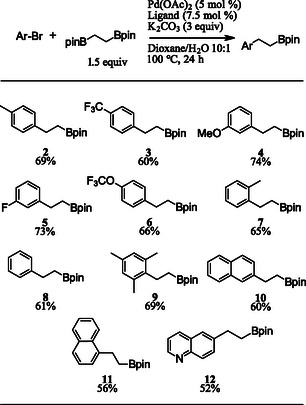
Aryl bromide substrate scope.

With optimized reaction conditions in hand, the substrate scope of aryl bromides was examined (**Scheme** **1**). A variety of 2‐, 3‐, and 4‐substituted aryl bromides are represented in the substrate scope with yields ranging from 60–74%. Electronically poor substrates were shown to give acceptable yields as seen in products **3** and **6**. Notably, the sterically hindered 2‐bromomesitylene was a good substrate, providing the desired product **9** in a 69% yield. A heterocyclic bromide was demonstrated in the reaction yielding product **12** in a 52% yield.

Further studies examining reactions with alkylbisboronic esters **13** and **14** with 3‐ and 4‐carbon chains were performed (**Table** **2**). It was found that the reaction worked best with a 2‐carbon linker under the optimized reaction conditions, as expected based on the favorable formation of a five‐membered oxygen‐bridged bisboronic ester intermediate.^[^ ^10^ ^]^ When changing the cosolvent to toluene, an inversion in yields was observed with the 4‐carbon alkylbisboronic ester **14** providing the highest yield of product **16** in a modest 38%.^[^ ^20^ ^]^

**Table 2:** Alkylbisboronic esters with varying chain lengths.

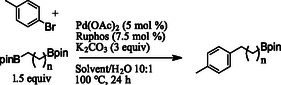		
Bisboronic Ester	Solvent = Dioxane	Solvent = Toluene
**1**, *n* = 1	**2**, 69%	**2**, 21%
**13**, *n* = 2	**15**, 25%	**15**, 22%
**14**, *n* = 3	**16**, 18%	**16**, 38%

**Scheme 2:**
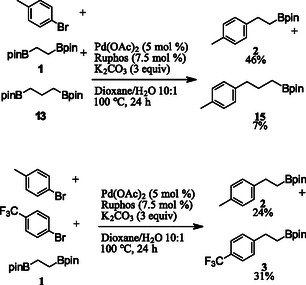
Competition experiments.

Several competition experiments were performed to better understand the mechanism of this reaction (**Scheme** **2**). A reaction with one equivalent of 4‐bromotoluene and the alkylbisboronic esters **1** and **13** provided 46% and 7% yields of product **2** and **15**, respectively. The faster formation of product **2** is consistent with that previously reported in alkylbisboronic ester cross‐couplings where a 5‐membered ring oxygen‐bridged bisboronic ester reacts faster than a similar 6‐membered ring intermediate.^[^ ^6^ ^]^ A second competition experiment examined the effects of the electronics of the aryl bromide substrate in a competition between 4‐bromotoluene and 4‐bromobenzotrifluoride. There was modest selectivity for the formation of the trifluoro‐substituted product **3** in this competition experiment which can be rationalized by a faster rate of oxidation addition of the electron‐poor aryl substrate.^[^ ^21^ ^]^

## 3 Conclusion

In conclusion, a method for the selective mono cross‐coupling of alkylbisboronic esters has been developed. A variety of aryl bromides were effective substrates in this reaction. It was determined that the reaction works best with an alkylbisboronic ester with a 2‐carbon linker over longer carbon chains. Further studies will be undertaken to improve the reaction efficiency of longer carbon chain linkers.

## 4 Experimental Section

4.1

4.1.1

##### General Methods

NMR spectra were recorded on a Bruker 400 MHz spectrometer. NMR data were collected at 25 °C. Proton chemical shifts were reported in ppm (*δ*) relative to the respective solvent resonance (CDCl_3_ *δ* 7.26). Data were reported as follows: proton chemical shift (multiplicity [singlet (s), doublet (d), triplet (t), quartet (q), multiplet (m)], coupling constants [Hz], integration). Carbon chemical shifts were reported in ppm (*δ*) relative to the respective solvent resonance (CDCl_3_, *δ* 77.16 ppm). 4,4,5,5‐Tetramethyl‐2‐[3‐(4,4,5,5‐tetramethyl‐1,3,2‐dioxaborolan‐2‐yl)propyl]‐1,3,2‐dioxaborolane (**13**) and 4,4,5,5‐Tetramethyl‐2‐[4‐(4,4,5,5‐tetramethyl‐1,3,2‐dioxaborolan‐2‐yl) butyl]‐1,3,2‐dioxaborolane (**14**) were prepared using known procedures.^[^ ^22^ ^]^ All monodentate ligands were converted to their corresponding HBF_4_ salts using a known procedure.^[^ ^23^ ^]^ All other reagents were commercially purchased and used as received.

##### Synthesis of 1,2‐Bis(4,4,5,5‐Tetramethyl‐1,3,2‐Dioxaborolan‐2‐Yl)ethane (1)

In a flame‐dried round bottom flask, bis(pinacolato)diboron (3.0 g, 12 mmol, 1.2 equiv), KO*t*Bu (1.4 g. 12 mmol, and 1.2 equiv), Xantphos (0.29 g, 0.5 mmol, and 0.050 equiv), CuCl (0.050 g, 0.5 mmol, and 0.050 equiv), and a magnetic stir bar was added. The reaction was then put under argon, and the reagents were dissolved in 50 mL of tetrahydrofuran and stirred for 10 min at 45 °C. Methanol (0.81 mL, 20 mmol, and 2.0 equiv), and 4,4,5,5‐tetramethyl‐2‐vinyl‐1,3,2‐dioxaborolane (1.7 mL, 10 mmol, and 1 equiv) were syringed into the flask, and the reaction continued to stir overnight at 45 °C. Upon completion, the product was filtered through celite, concentrated under reduced pressure, and purified via column chromatography in 92:8 hexanes/EtOAc to afford 2.2 g (79%) of **1** as a clear oil. The characterization data was consistent with that previously reported.^[^ ^24^ ^]^

##### General Procedure

In a 10 mL high pressure vessel, 1,2‐bis(4,4,5,5‐tetramethyl‐1,3,2‐dioxaborolan‐2‐yl)ethane (**1**) (85 mg, 0.30 mmol, and 1.5 equiv), aryl bromide (0.20 mmol, 1.0 equiv), palladium (II) acetate (2.3 mg, 0.010 mmol, and 0.050 equiv), RuPhos‐HBF_4_ (8.3 mg, 0.015 mmol, and 0.075 equiv), potassium carbonate (83 mg, 0.60 mmol, and 3.0 equiv), and a magnetic stir bar was added. The reagents were dissolved in 1,4‐dioxane (1 mL) and degassed H_2_O (0.1 mL). The vessel was then purged with argon, quickly sealed, and stirred at 100 °C for 24 h. Upon completion, the reaction was extracted with 3 × 10 mL of ethyl acetate and 10 mL of water. The organic layer was dried with Na_2_SO_4_ and concentrated under reduced pressure. The mixture was then purified via column chromatography in 98:2 hexanes/EtOAc.

## Supporting Information

The authors have cited additional references within the Supporting Information.^[^^25–28^^]^

## Conflict of Interest

The authors declare no conflict of interest.

## Supporting information

Supplementary Material

## Data Availability

The data that support the findings of this study are available in the supplementary material of this article.
