# Supplementary material for: Pd‐Catalyzed Cross‐Coupling of Alkylbisboronic Esters
Source: ChemistryOpen. 2025 Apr 10;14(10):e202500195. doi: 10.1002/open.202500195 (PMC12518026; doi:10.1002/open.202500195)
Supplement: Supplementary file 1 — Supplementary Material [file OPEN-14-e202500195-s001.pdf]

Table of Contents

Competition Experiment Procedures (S2)
Characterization Data (S2)
References (S5)
^{1}H and ^{13}C NMR Spectra (S6)

Competition Experiments: In a 10 mL high pressure vessel, 1,2-bis(4,4,5,5-tetramethyl-1,3,2-dioxaborolan-2-yl)ethane (**1**) (0.20 mmol, 1.0 equiv), 4,4,5,5-Tetramethyl-2-[3-(4,4,5,5-tetramethyl-1,3,2-dioxaborolan-2-yl)propyl]-1,3,2-dioxaborolane (**13**) (0.20 mmol, 1.0 equiv), 4-bromotoluene (0.20 mmol, 1.0 equiv), palladium (II) acetate (2.3 mg, 0.010 mmol, 0.050 equiv), RuPhos-HBF_4 (8.3 mg, 0.015 mmol, 0.075 equiv), potassium carbonate (83 mg, 0.60 mmol, 3.0 equiv), and a magnetic stir bar was added. The reagents were dissolved in 1,4-dioxane (1 mL) and degassed H_2O (0.1 mL). The vessel was then purged with argon, quickly sealed, and stirred at 100 °C for 24 hours. Upon completion, the reaction was extracted with 3 x 10 mL of ethyl acetate and 10 mL of water. The organic layer was dried with Na_2SO_4 and concentrated under reduced pressure. 1,4-dimethoxylbenzene (0.20 mmol) was added as an internal standard and the yields were determined by ^{1}H NMR of the unpurified reaction mixture.

In a 10 mL high pressure vessel, 1,2-bis(4,4,5,5-tetramethyl-1,3,2-dioxaborolan-2-yl)ethane (**1**) (57 mg, 0.20 mmol, 1.0 equiv), 4-bromotoluene (0.20 mmol, 1.0 equiv), 4-bromobenzotrifluoride (0.20 mmol, 1.0 equiv), palladium (II) acetate (2.3 mg, 0.010 mmol, 0.050 equiv), RuPhos-HBF_4 (8.3 mg, 0.015 mmol, 0.075 equiv), potassium carbonate (83 mg, 0.60 mmol, 3.0 equiv), and a magnetic stir bar was added. The reagents were dissolved in 1,4-dioxane (1 mL) and degassed H_2O (0.1 mL). The vessel was then purged with argon, quickly sealed, and stirred at 100 °C for 24 hours. Upon completion, the reaction was extracted with 3 x 10 mL of ethyl acetate and 10 mL of water. The organic layer was dried with Na_2SO_4 and concentrated under reduced pressure. 1,4-dimethoxylbenzene (0.20 mmol) was added as an internal standard and the yields were determined by ^{1}H NMR of the unpurified reaction mixture.

Characterization Data of Products:

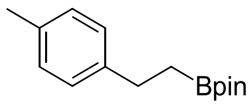

4,4,5,5-Tetramethyl-2-[2-(*p*-tolyl)ethyl]-1,3,2-dioxaborolane (**2**)[25] was prepared according to the general procedure and isolated as a colorless oil 34 mg (69% yield). ^{1}H NMR (400 MHz, Chloroform-*d*) δ 7.13 – 7.06 (m, 4H), 2.72 (t, *J* = 8.2 Hz, 2H), 2.31 (s, 3H), 1.24 (s, 12H), 1.13 (t, *J* = 8.2 Hz, 2H); ^{13}C NMR (101 MHz, Chloroform-*d*) δ 141.54, 134.98, 129.00, 127.98, 83.21, 29.65, 24.95, 21.10.

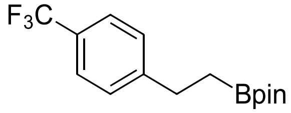

4,4,5,5-Tetramethyl-2-{2-[*p*-(trifluoromethyl)phenyl]ethyl}-1,3,2-dioxaborolane (**3**)[25] was prepared according to the general procedure and isolated as a colorless oil 36 mg (60% yield). ^{1}H NMR (400 MHz, Chloroform-*d*) δ 7.51 (d, *J* = 7.7 Hz, 2H), 7.32 (d, *J* = 7.9 Hz, 2H), 2.80 (t, *J* = 8.1 Hz, 2H), 1.21 (s, 12H), 1.15 (t, *J* = 8.1 Hz, 2H); ^{13}C NMR (101 MHz, Chloroform-*d*) δ 148.65, 128.46, 128.07 (q, *J* = 33 Hz), 125.25 (q, *J* = 4 Hz), 124.59 (q, *J* = 272 Hz), 83.40, 29.99, 24.94.

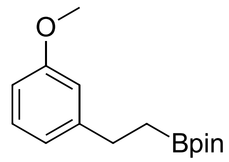

2-[2-(*m*-Methoxyphenyl)ethyl]-4,4,5,5-tetramethyl-1,3,2-dioxaborolane (**4**)[26] was prepared according to the general procedure and isolated as a colorless oil 39 mg (74% yield). ^{1}H NMR (400 MHz, Chloroform-*d*) δ 7.17 (t, *J* = 7.8 Hz, 1H), 6.81 (d, *J* = 7.5 Hz, 1H), 6.78 (t, *J* = 2.1 Hz, 1H), 6.71 (dd, *J* = 7.8, 2.1 Hz, 1H), 3.79 (s, 3H), 2.73 (t, *J* = 8.2 Hz, 2H), 1.23 (s, 12H), 1.14 (t, *J* = 8.2 Hz, 2H); ^{13}C NMR (101 MHz, Chloroform-*d*) δ 159.70, 146.26, 129.26, 120.56, 113.77, 111.14, 83.24, 55.23, 30.17, 24.96.

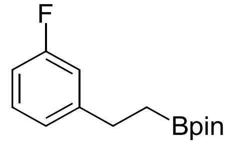

2-[2-(*m*-Fluorophenyl)ethyl]-4,4,5,5-tetramethyl-1,3,2-dioxaborolane (**5**)[26] was prepared according to the general procedure and isolated as a colorless oil 37 mg (73% yield). ^{1}H NMR (400 MHz, Chloroform-*d*) δ 7.20 (td, *J* = 7.9, 6.1 Hz, 1H), 6.98 (d, *J* = 7.6 Hz, 1H), 6.92 (dt, *J* = 10.3, 2.1 Hz, 1H), 6.84 (td, *J* = 8.0, 1.7 Hz, 1H), 2.74 (t, *J* = 8.1 Hz, 2H), 1.22 (s, 12H), 1.13 (t, *J* = 8.1 Hz, 2H); ^{13}C NMR (101 MHz, Chloroform-*d*) δ 163.02 (d, *J* = 245 Hz), 147.17 (d, *J* = 7 Hz), 129.65 (d, *J* = 8 Hz), 123.79 (d, *J* = 3 Hz), 115.00 (d, *J* = 21 Hz), 112.45 (d, *J* = 21 Hz), 83.33, 29.87 (d, *J* = 2 Hz), 24.94.

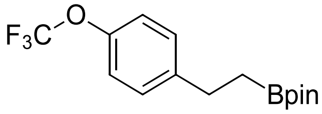

4,4,5,5-Tetramethyl-2-[2-(*p*-trifluoromethoxyphenyl)ethyl]-1,3,2-dioxaborolane (**6**)[27] was prepared according to the general procedure and isolated as a colorless oil 42 mg (66% yield). ^{1}H NMR (400 MHz, Chloroform-*d*) δ 7.22 (d, *J* = 8.6 Hz, 2H), 7.10 (d, *J* = 7.8 Hz, 2H), 2.75 (t, *J* = 8.1 Hz, 2H), 1.20 (s, 12H), 1.13 (t, *J* = 8.1 Hz, 2H); ^{13}C NMR (101 MHz, Chloroform-*d*) δ 147.34 (q, *J* = 2 Hz), 143.25, 129.39, 120.90, 120.69 (q, *J* = 256 Hz), 83.34, 29.44, 24.91.

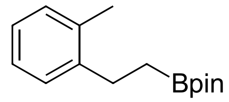

4,4,5,5-Tetramethyl-2-[2-(*o*-tolyl)ethyl]-1,3,2-dioxaborolane (**7**)[25] was prepared according to the general procedure and isolated as a colorless oil 32 mg (65% yield). ^{1}H NMR (400 MHz, $CDCl_3$) δ 7.19 – 7.16 (m, 1H), 7.13 – 7.04 (m, 3H), 2.71 (t, *J* = 8.1 Hz, 2H), 2.31 (s, 3H), 1.23 (s, 12H), 1.10 (t, *J* = 8.1 Hz, 2H); ^{13}C NMR (101 MHz, $CDCl_3$) δ 142.64, 135.90, 130.08, 128.20, 125.97, 125.74, 83.22, 27.29, 24.95, 19.40.

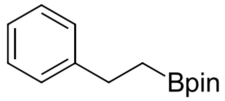

4,4,5,5-Tetramethyl-2-phenethyl-1,3,2-dioxaborolane (**8**)[25] was prepared according to the general procedure and isolated as a colorless oil 28 mg (61% yield). ^{1}H NMR (400 MHz, Chloroform-*d*) δ 7.28 – 7.21 (m, 4H), 7.17 – 7.13 (m, 1H) 2.75 (t, *J* = 8.1 Hz, 2H), 1.22 (s, 12H), 1.14 (t, *J* = 8.1 Hz, 2H); ^{13}C NMR (101 MHz, $CDCl_3$) δ 144.57, 128.32, 128.15, 125.64, 83.24, 30.10, 24.95.

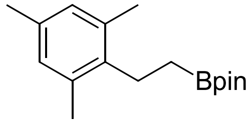

4,4,5,5-tetramethyl-2-(2,4,6-trimethylphenethyl)-1,3,2-dioxaborolane (**9**)[26] was prepared according to the general procedure and isolated as a colorless oil 38 mg (69% yield). ^{1}H NMR (400 MHz, Chloroform-*d*) δ 6.83 (s, 2H), 2.71 – 2.66 (m, 2H), 2.31 (s, 6H), 2.25 (s, 3H), 1.29 (s, 12H), 0.99 – 0.95 (m, 2H); ^{13}C NMR (101 MHz, $CDCl_3$) δ 138.60, 135.74, 134.75, 128.92, 83.20, 24.97, 23.36, 20.91, 19.77.

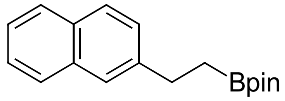

4,4,5,5-Tetramethyl-2-[2-(2-naphthyl)ethyl]-1,3,2-dioxaborolane (**10**)[25] was prepared according to the general procedure and isolated as a colorless oil 34 mg (60% yield). ^{1}H NMR (400 MHz, Chloroform-*d*) δ 7.81 – 7.75 (m, 3H), 7.66 (s, 1H), 7.46 – 7.37 (m, 3H), 2.93 (t, *J* = 8.1 Hz, 2H), 1.25 (t, *J* = 8.1 Hz, 2H), 1.23 (s, 12H); ^{13}C NMR (101 MHz, $CDCl_3$) δ 142.12, 133.80, 132.06, 127.82, 127.70, 127.57, 127.42, 125.85, 125.83, 125.05, 83.29, 30.27, 24.97.

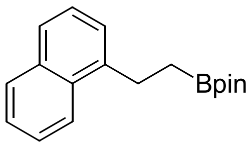

4,4,5,5-Tetramethyl-2-[2-(1-naphthyl)ethyl]-1,3,2-dioxaborolane (**11**)[26] was prepared according to the general procedure and isolated as a colorless oil 32 mg (56% yield). ^{1}H NMR (400 MHz, Chloroform-*d*) δ 8.10 (d, *J* = 8.2 Hz, 1H), 7.85 (dd, *J* = 8.1, 1.4 Hz, 1H), 7.74 – 7.65 (m, 1H), 7.52 – 7.44 (m, 2H), 7.41 – 7.37 (m, 2H), 3.22 (t, *J* = 8.1 Hz, 2H), 1.30 (t, *J* = 8.1 Hz, 2H), 1.25 (s, 12H); ^{13}C NMR (101 MHz, $CDCl_3$) δ 140.60, 133.95, 131.90, 128.78, 126.45, 125.72, 125.68, 125.46, 125.13, 124.10, 83.29, 27.08, 24.99.

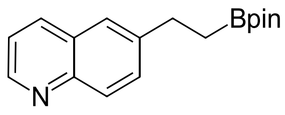

4,4,5,5-Tetramethyl-2-[2-(7-quinolyl)ethyl]-1,3,2-dioxaborolane (**12**) was prepared according to the general procedure and isolated as a colorless oil 29 mg (52% yield). ^{1}H NMR (400 MHz,

$CDCl_3$) δ 8.83 – 8.82 (m, 1H), 8.06 (d, *J* = 7.8 Hz, 1H), 7.99 (d, *J* = 9.2 Hz, 1H), 7.61 – 7.56 (m, 2H), 7.33 (dd, *J* = 8.3, 4.2 Hz, 1H), 2.93 (t, *J* = 8.0 Hz, 2H), 1.23 (t, *J* = 8.0 Hz, 2H), 1.19 (s, 12H). ^{13}C NMR (101 MHz, $CDCl_3$) δ 149.58, 147.18, 142.96, 135.68, 131.07, 129.18, 128.41, 125.56, 121.07, 83.31, 30.04, 24.91. HRMS-ESI (*m/z*) $[M+1]^+$ calcd for $C_{17}H_{23}BNO_2$, 284.1816; found, 284.1809.

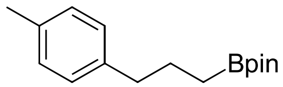

4,4,5,5-tetramethyl-2-(3-(p-tolyl)propyl)-1,3,2-dioxaborolane (**15**)[28] was prepared according to the general procedure and isolated as a colorless oil 13 mg (25% yield). ^{1}H NMR (400 MHz, Chloroform-*d*) δ 7.07 (s, 4H), 2.57 (t, *J* = 7.8 Hz, 2H), 2.31 (s, 3H), 1.71 (p, *J* = 7.8 Hz, 2H), 1.24 (s, 12H), 0.82 (t, *J* = 7.9 Hz, 2H); ^{13}C NMR (101 MHz, Chloroform-*d*) δ 139.78, 135.08, 129.00, 128.57, 83.06, 38.29, 26.36, 24.98, 21.12.

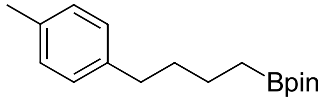

4,4,5,5-Tetramethyl-2-[4-(*p*-tolyl)butyl]-1,3,2-dioxaborolane (**16**) was prepared according to the general procedure except using 10:1 toluene/H_2O for the solvent and isolated as a colorless oil 21 mg (38% yield). ^{1}H NMR (400 MHz, $CDCl_3$) δ 7.07 (s, 4H), 2.56 (t, *J* = 7.6 Hz, 2H), 2.31 (s, 3H), 1.64 – 1.57 (m, 2H), 1.46 (p, *J* = 7.4 Hz, 2H), 1.24 (s, 12H), 0.81 (t, *J* = 7.7 Hz, 2H); ^{13}C NMR (101 MHz, $CDCl_3$) δ 140.00, 135.02, 129.03, 128.41, 83.04, 35.46, 34.47, 24.98, 23.92, 21.12; HRMS-MALDI (AgNP matrix) (*m/z*) $[M+Ag]^+$ calcd for $C_{17}H_{27}AgBO_2$, 381.1150; found, 381.1128.

References:

[25] J. Liu, J. Du, F. Yu, L. Gan, G. Liu, Z. Huang, *ACS Catal.* **2023**, *13,* 7995–8003.

[26] A. Fu, L. Zhao, C. Li, M. Luo, X. Zeng, *Organometallics* **2021**, *40*, 2204–2208.

[27] J. Hu, G. Wang, S. Li, Z. Shi, *Angew. Chem. Int. Ed.* **2018**, *57*, 15227–15231.

[28] P. Huninik, J. Szyling, A. Czapik, J. Walkowiak, *Green Chem.* **2023**, *25*, 3715–3722.

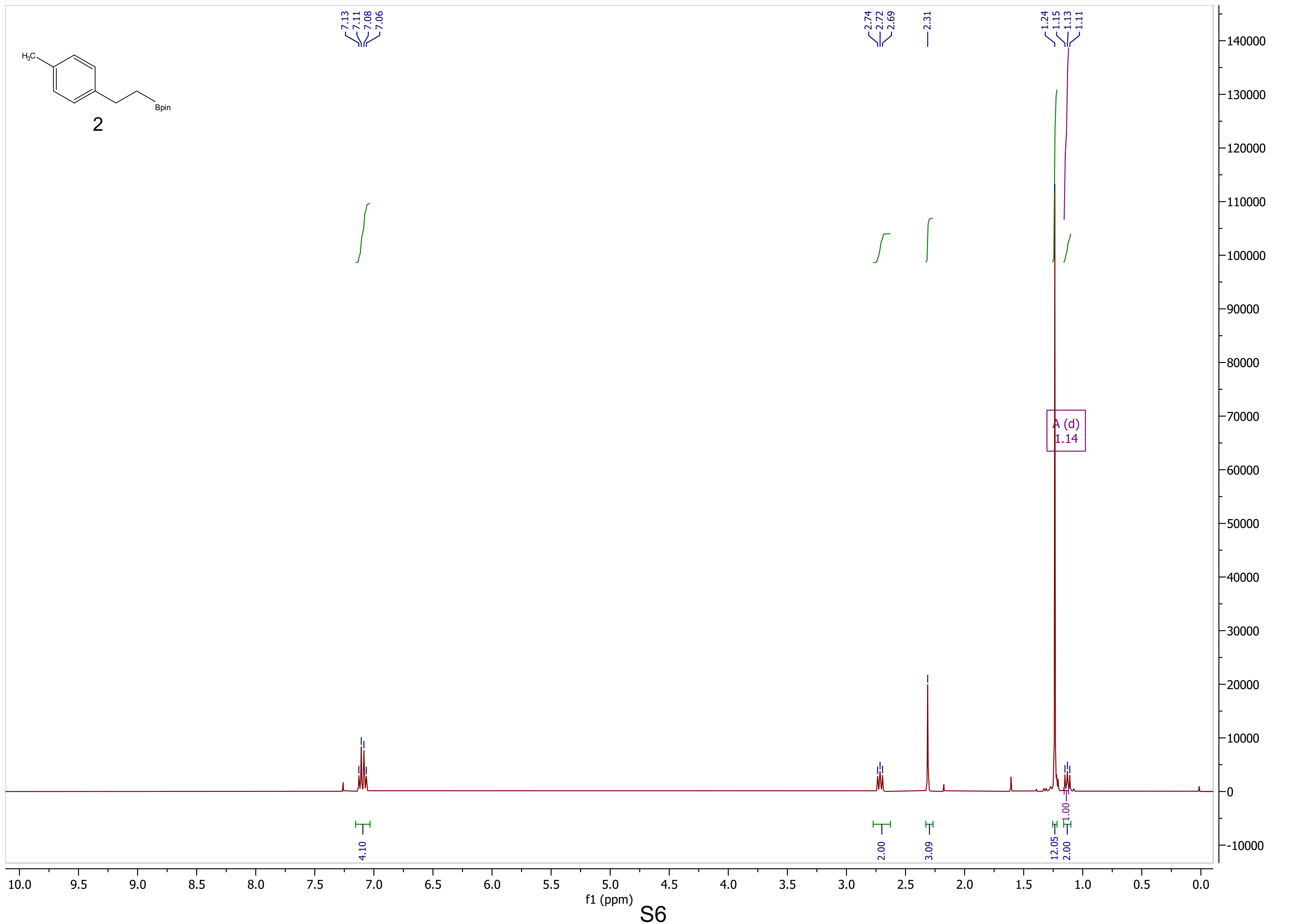
H3C
Bpin
2
7.13
7.11
7.08
7.06
2.74
2.72
2.69
2.31
1.24
1.15
1.13
1.11
A (d)
1.14
4.10
2.00
3.09
12.05
2.00
1.00
f1 (ppm)

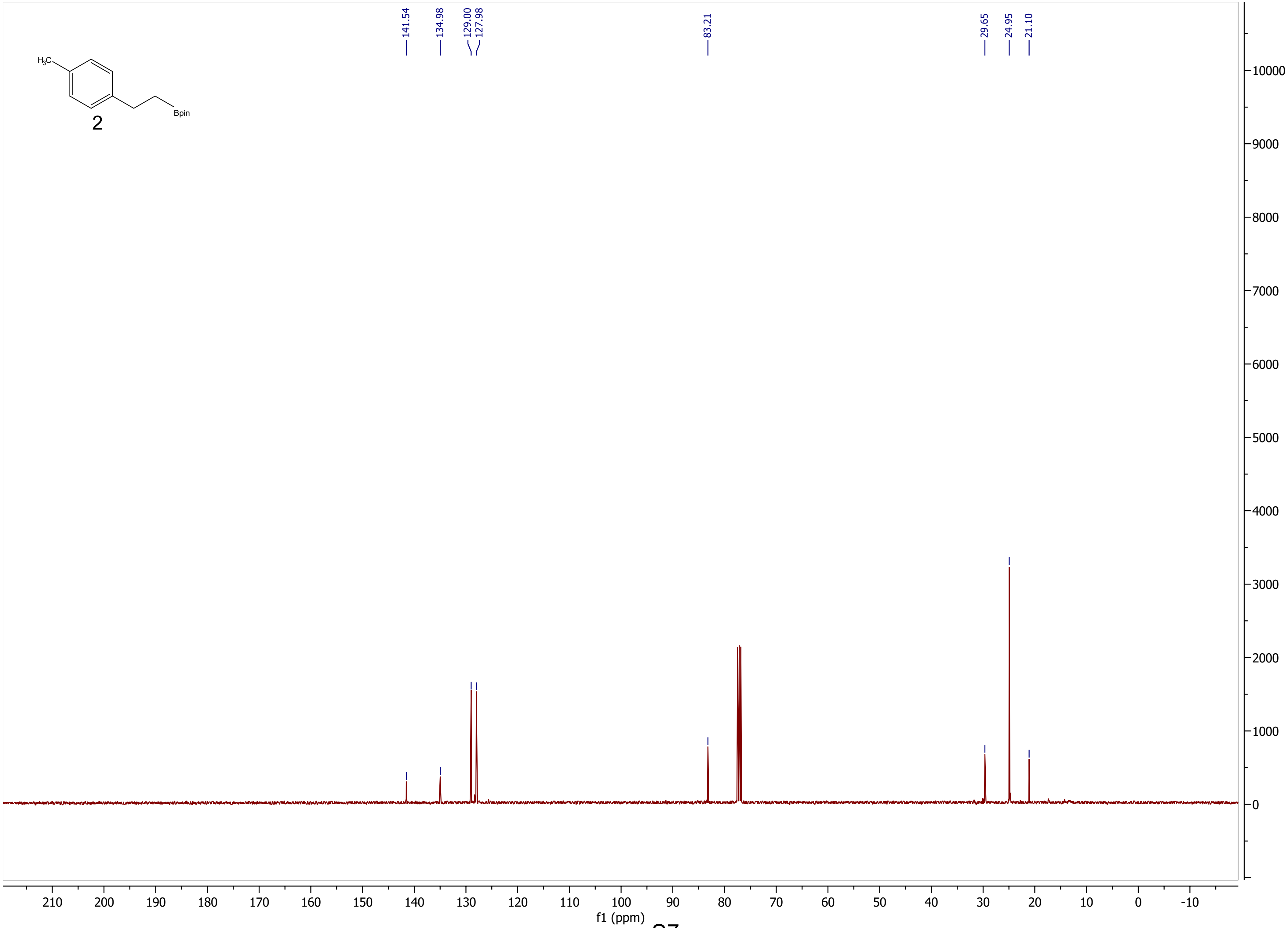
141.54
134.98
129.00
127.98
83.21
29.65
24.95
21.10
H3C
Bpin
2
10000
9000
8000
7000
6000
5000
4000
3000
2000
1000
0
210
200
190
180
170
160
150
140
130
120
110
100
90
80
70
60
50
40
30
20
10
0
-10
f1 (ppm)

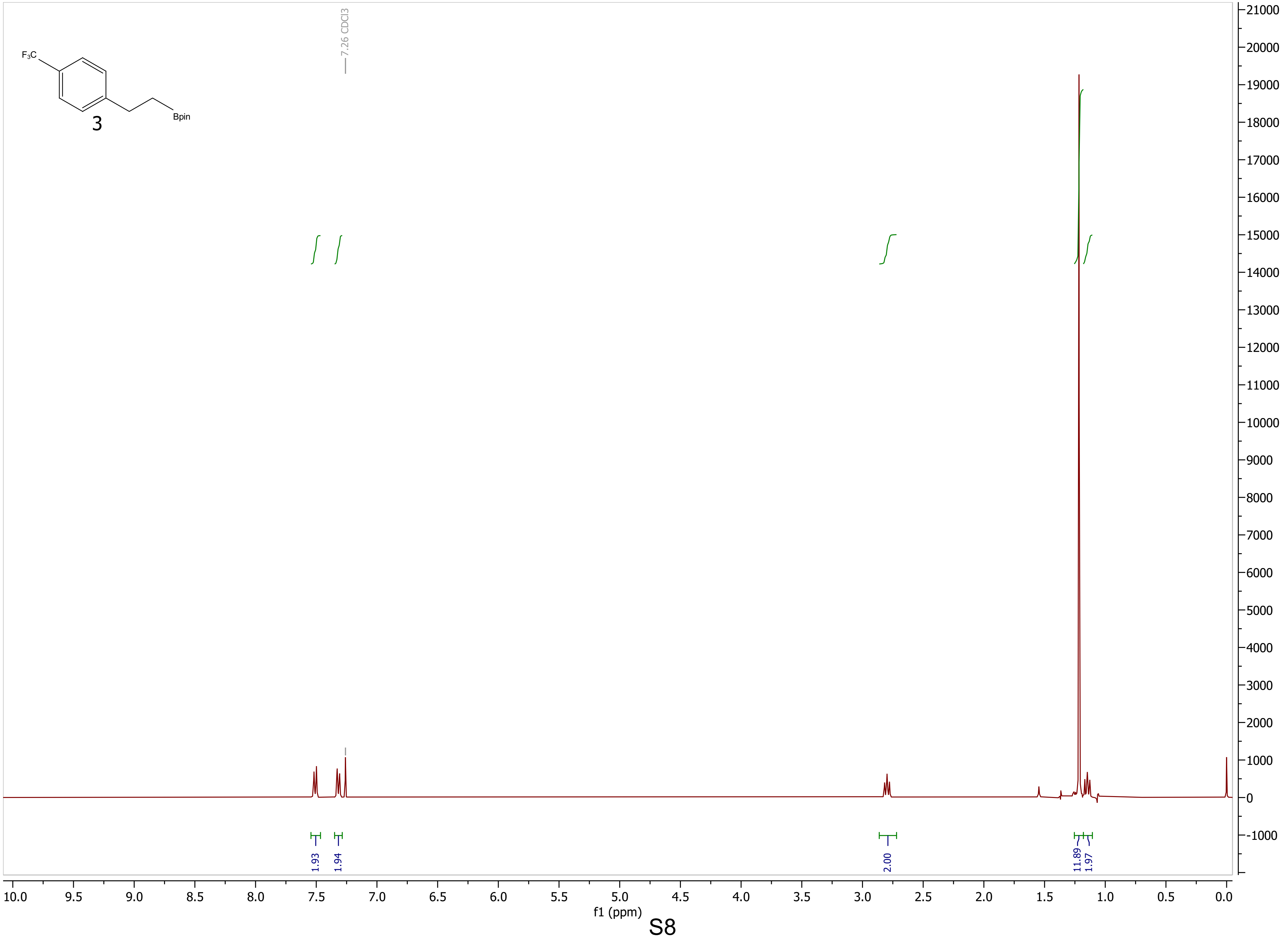

F3C
3
Bpin
7.26 CDCl3
1.93
1.94
2.00
11.89
1.97
f1 (ppm)

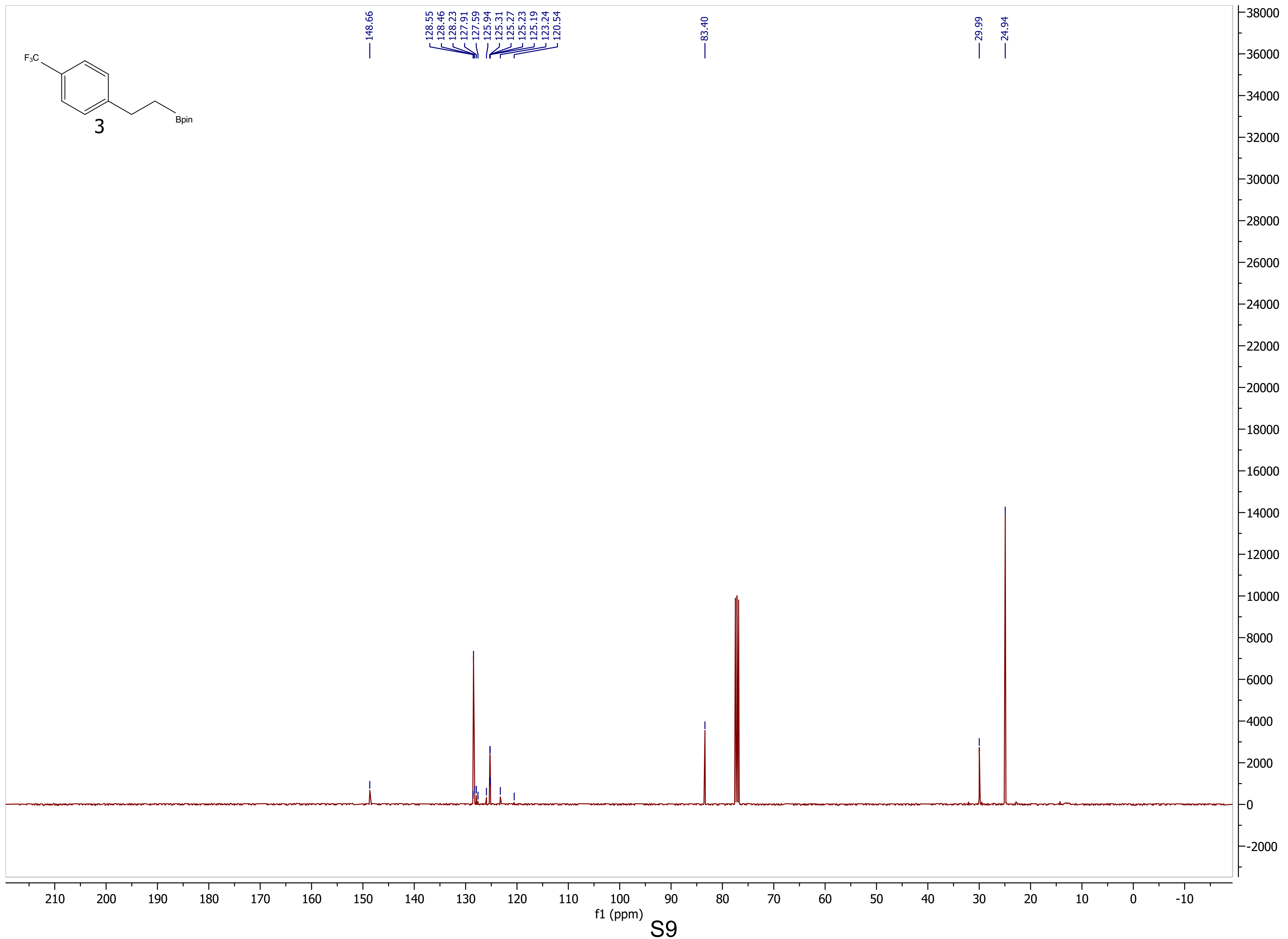

F3C
Bpin
3
148.66
128.55
128.46
128.23
127.91
127.59
125.94
125.31
125.27
125.23
125.19
123.24
120.54
83.40
29.99
24.94
f1 (ppm)

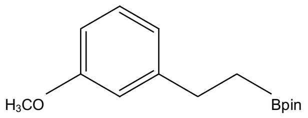

4

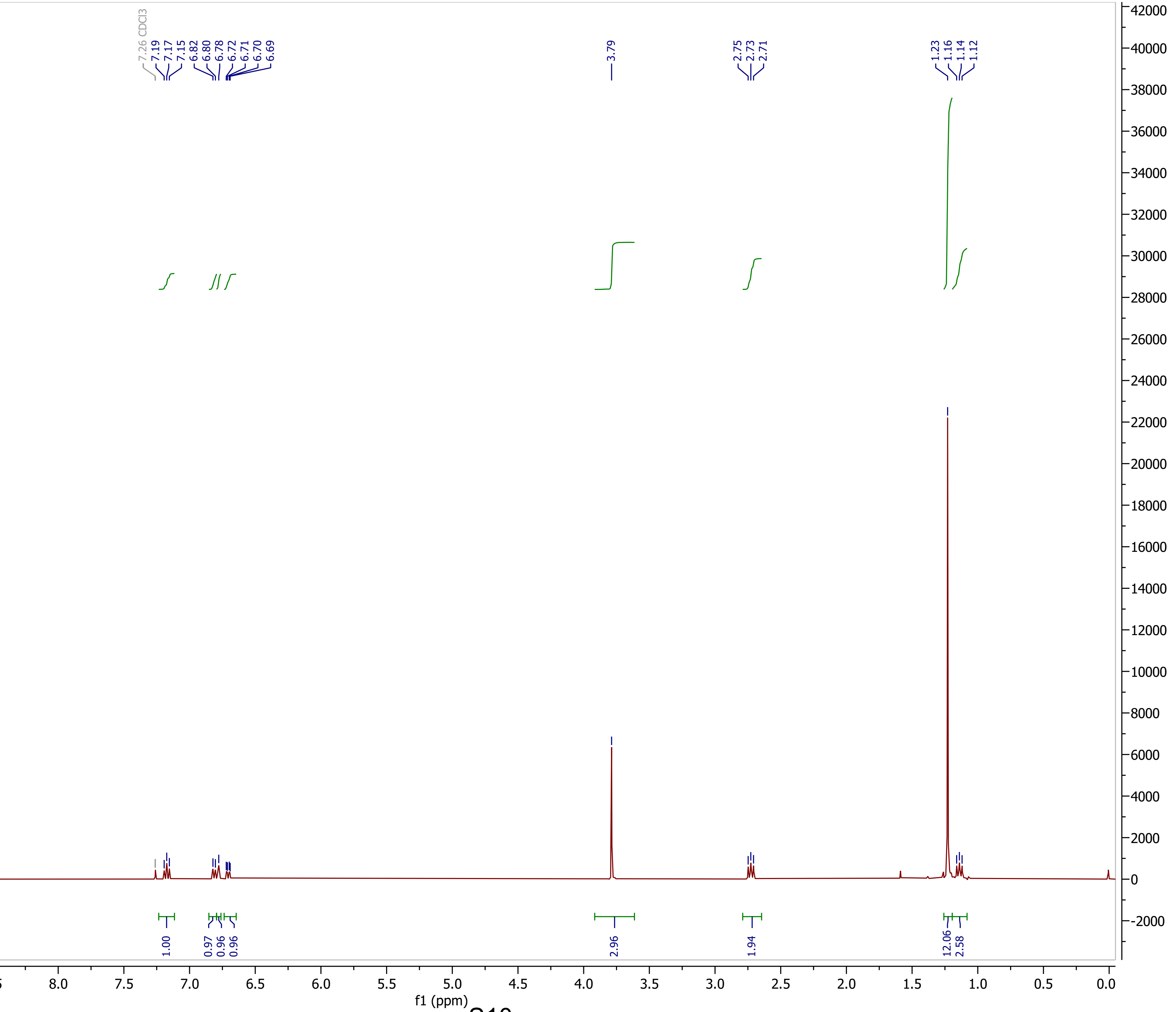
7.26 CDCl3
7.19
7.17
7.15
6.82
6.80
6.78
6.72
6.71
6.70
6.69
3.79
2.75
2.73
2.71
1.23
1.16
1.14
1.12
1.00
0.97
0.96
0.96
2.96
1.94
12.06
2.58
f1 (ppm)

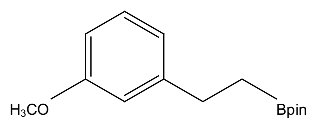
H3CO
Bpin

4

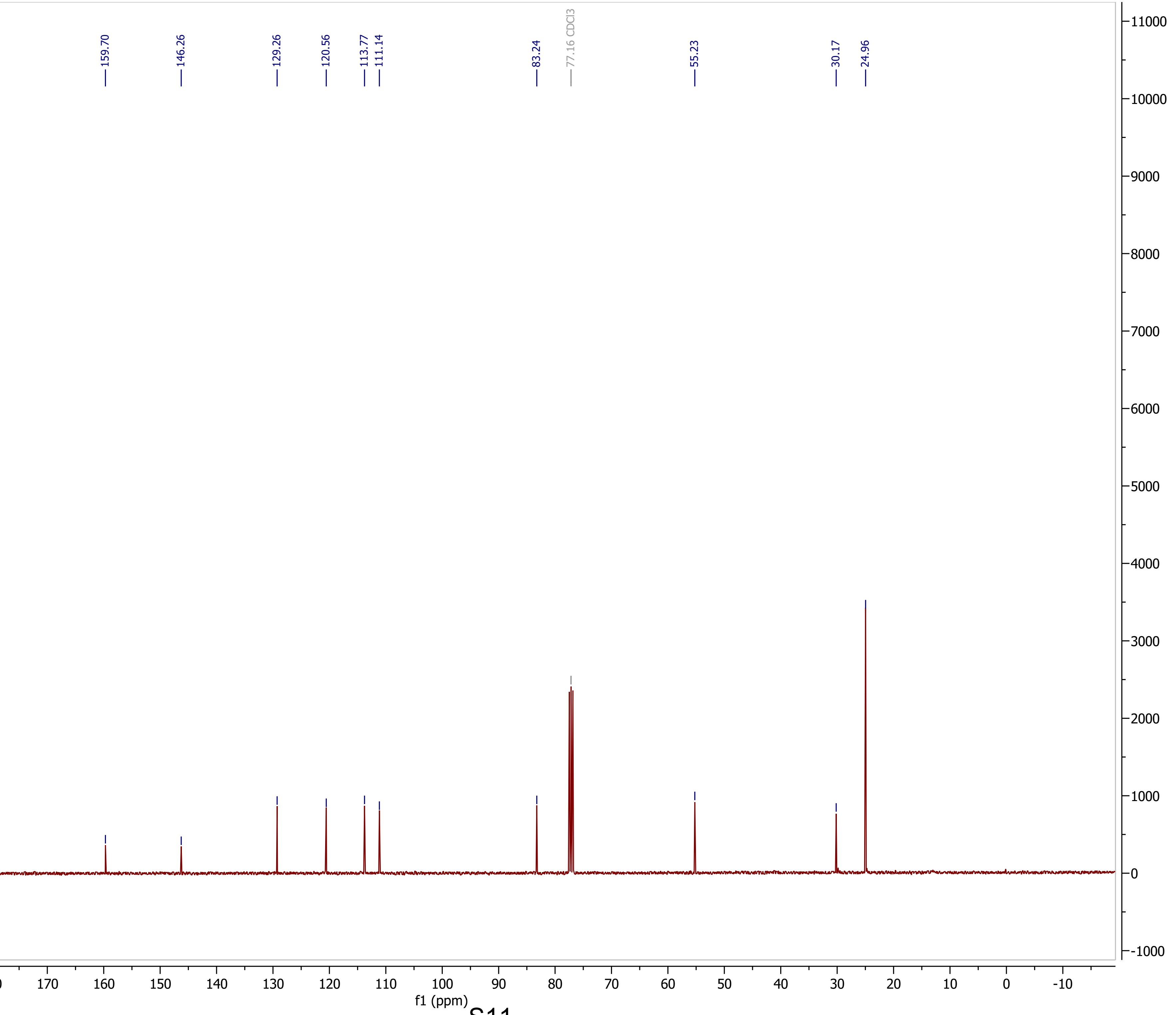
159.70
146.26
129.26
120.56
113.77
111.14
83.24
77.16 CDCl3
55.23
30.17
24.96
f1 (ppm)

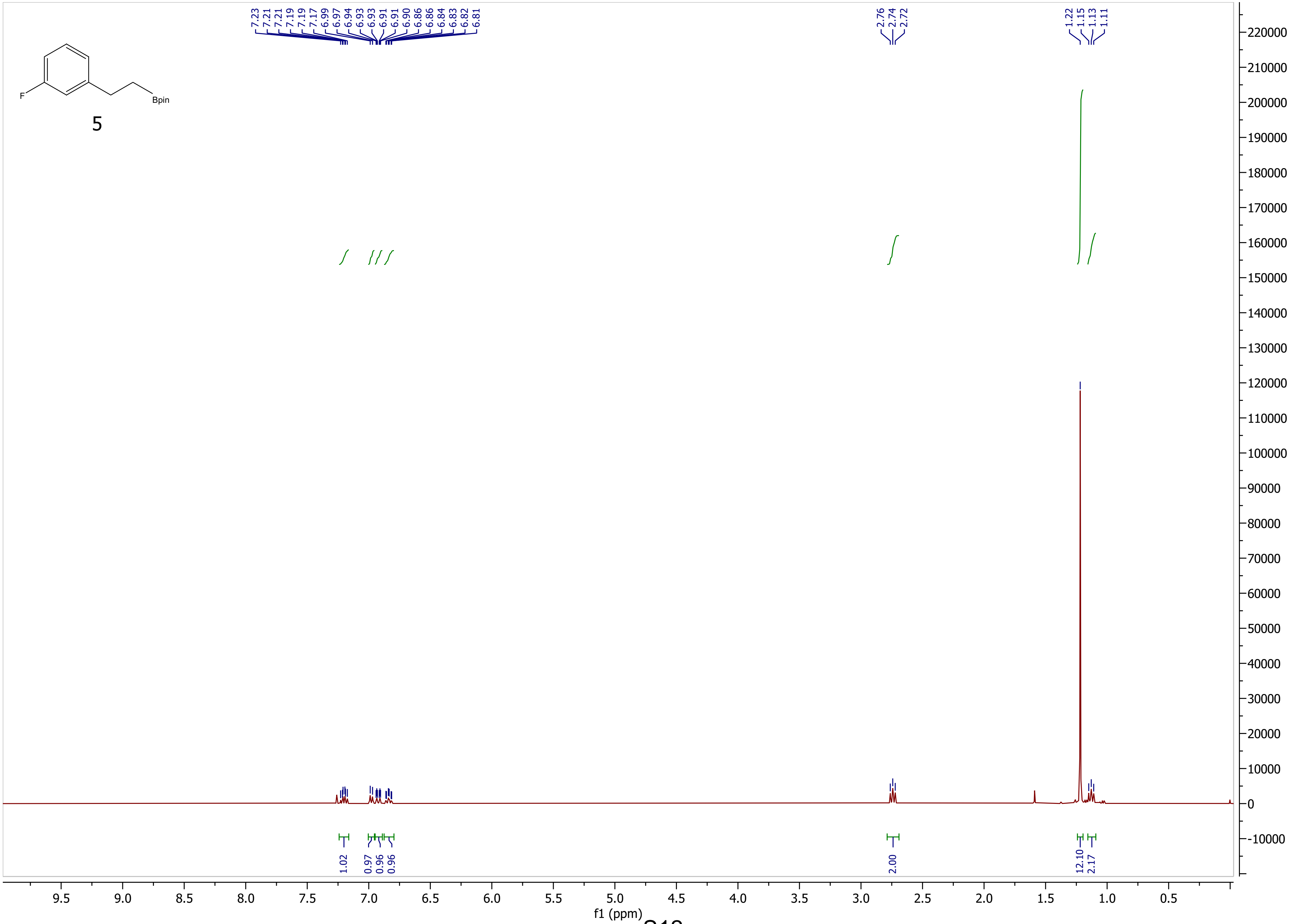
F
Bpin
5
7.23
7.21
7.21
7.19
7.19
7.17
6.99
6.97
6.94
6.93
6.93
6.91
6.91
6.90
6.86
6.86
6.84
6.83
6.82
6.81
2.76
2.74
2.72
1.22
1.15
1.13
1.11
1.02
0.97
0.96
0.96
2.00
12.10
2.17
f1 (ppm)

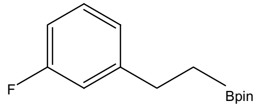
F
Bpin

5

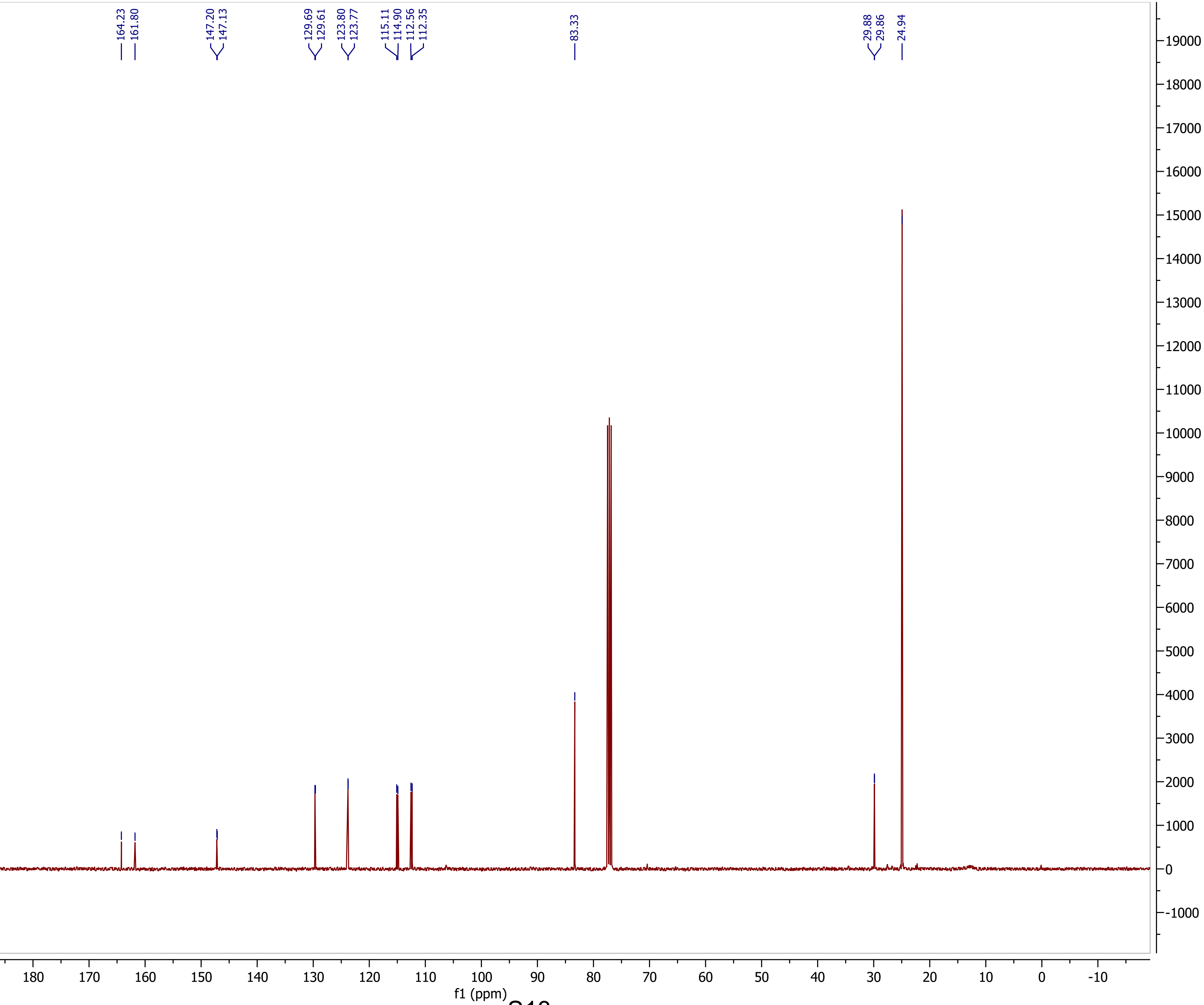
164.23
161.80
147.20
147.13
129.69
129.61
123.80
123.77
115.11
114.90
112.56
112.35
83.33
29.88
29.86
24.94
19000
18000
17000
16000
15000
14000
13000
12000
11000
10000
9000
8000
7000
6000
5000
4000
3000
2000
1000
0
-1000
210
200
190
180
170
160
150
140
130
120
110
100
90
80
70
60
50
40
30
20
10
0
-10
f1 (ppm)

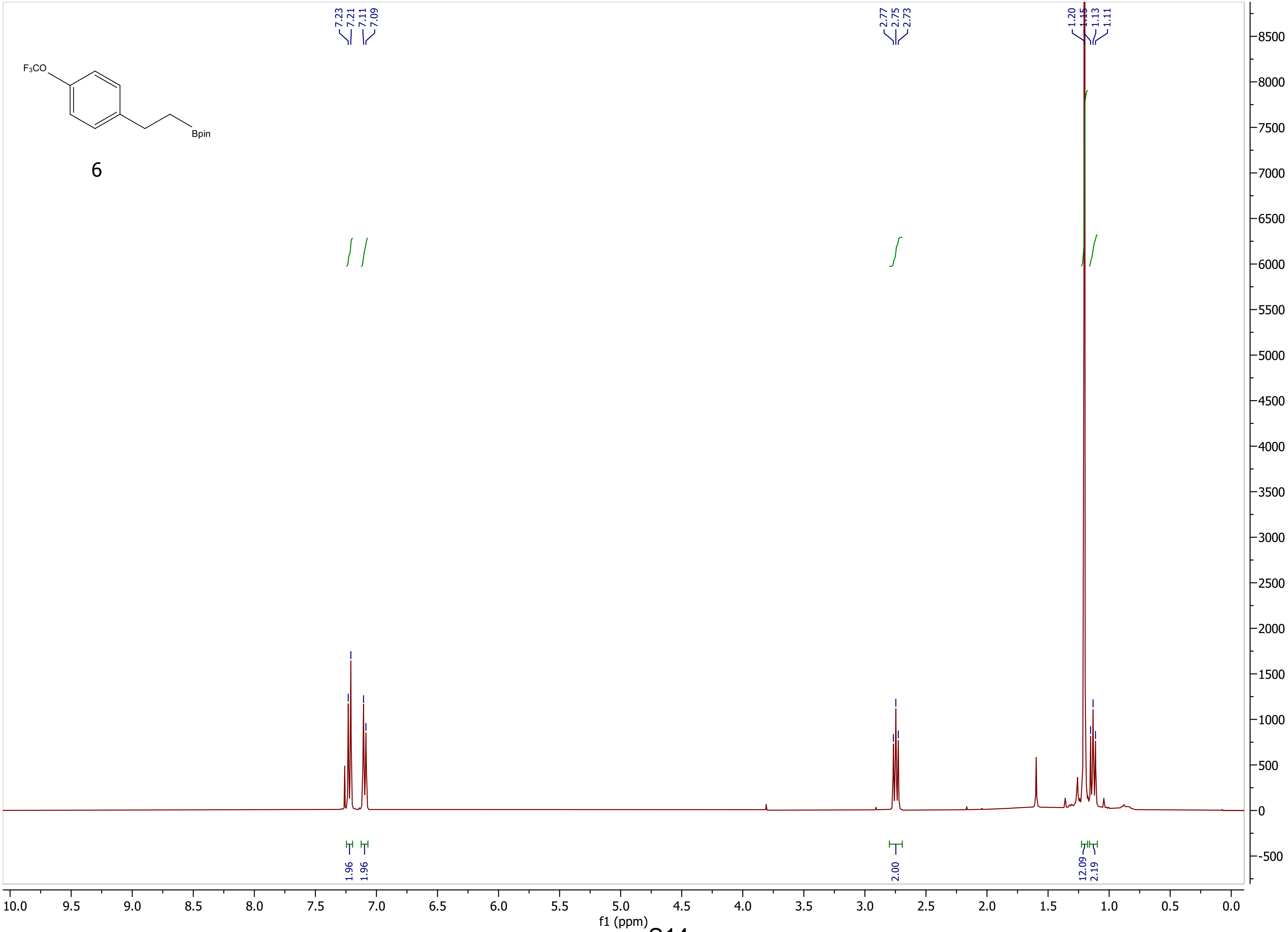

F3CO
Bpin
7.23
7.21
7.11
7.09
2.77
2.75
2.73
1.20
1.15
1.13
1.11
1.96
1.96
2.00
12.09
2.19
f1 (ppm)

6

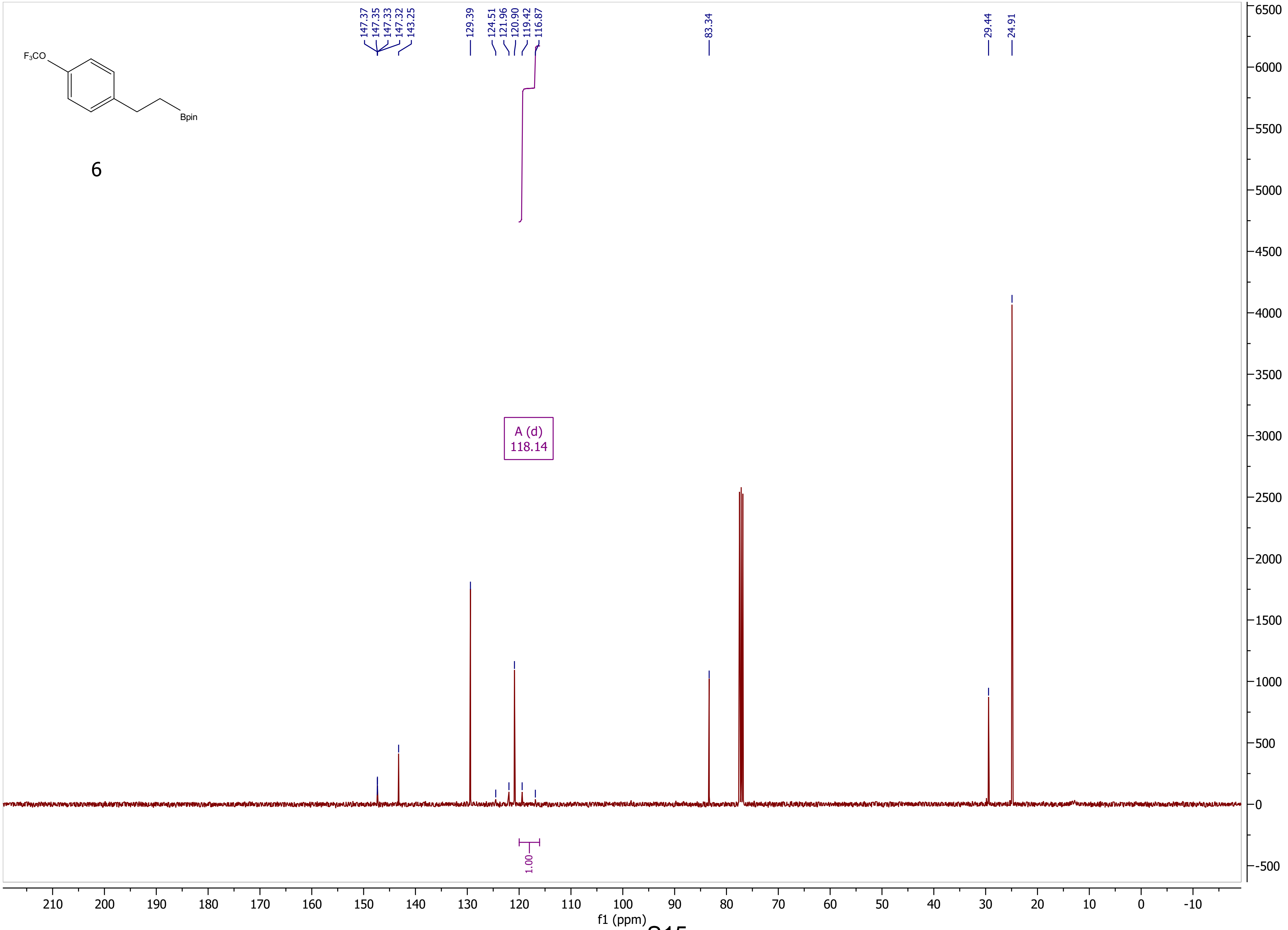
F3CO
Bpin
6
147.37
147.35
147.33
147.32
143.25
129.39
124.51
121.96
120.90
119.42
116.87
83.34
29.44
24.91
A (d)
118.14
1.00
f1 (ppm)

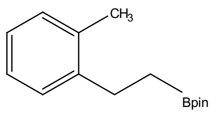
CH3
Bpin

7

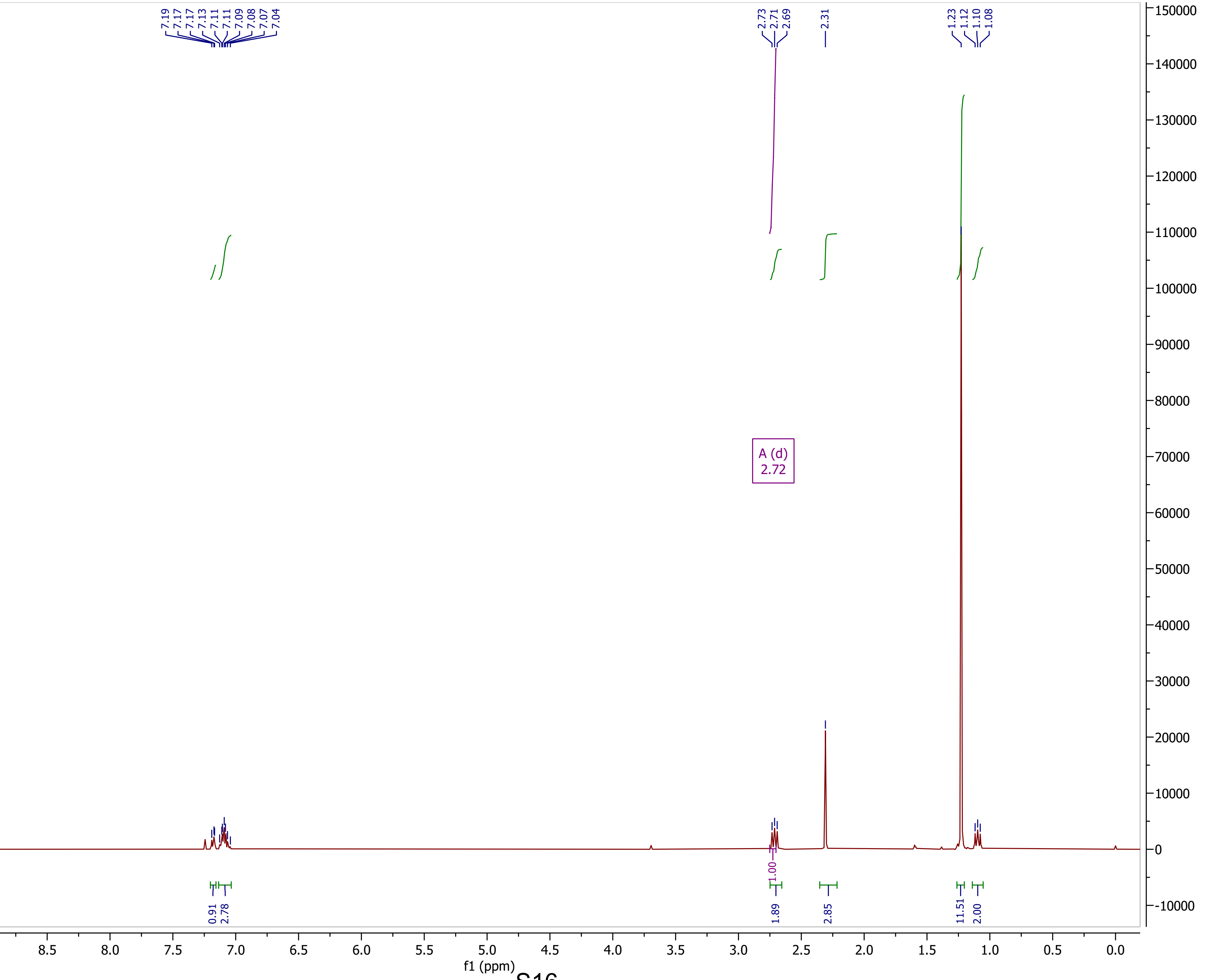
7.19
7.17
7.17
7.13
7.11
7.11
7.09
7.08
7.07
7.04
2.73
2.71
2.69
2.31
1.23
1.12
1.10
1.08
A (d)
2.72
1.00
0.91
2.78
1.89
2.85
11.51
2.00
f1 (ppm)

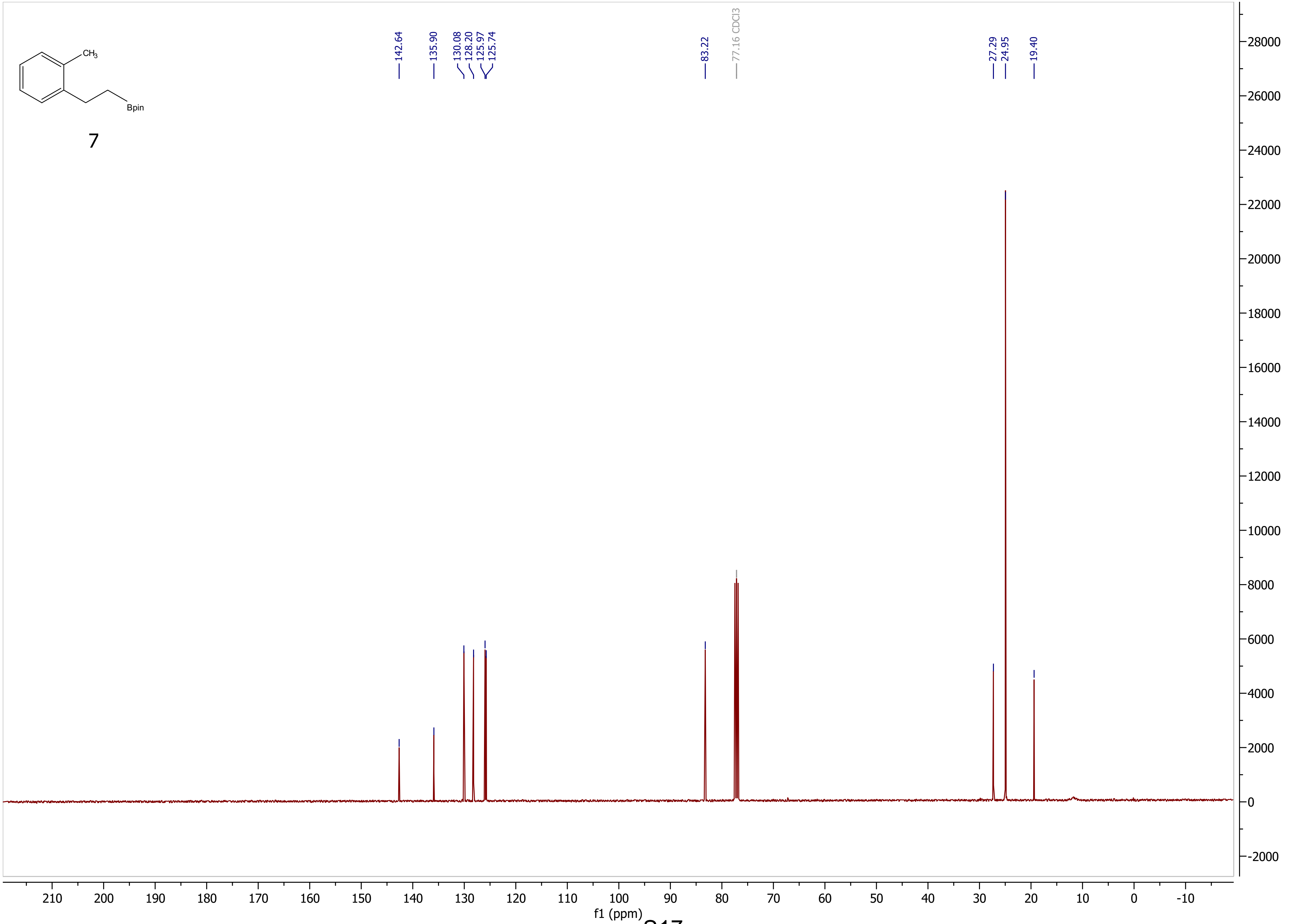
CH3
Bpin
7
142.64
135.90
130.08
128.20
125.97
125.74
83.22
77.16 CDCl3
27.29
24.95
19.40
f1 (ppm)

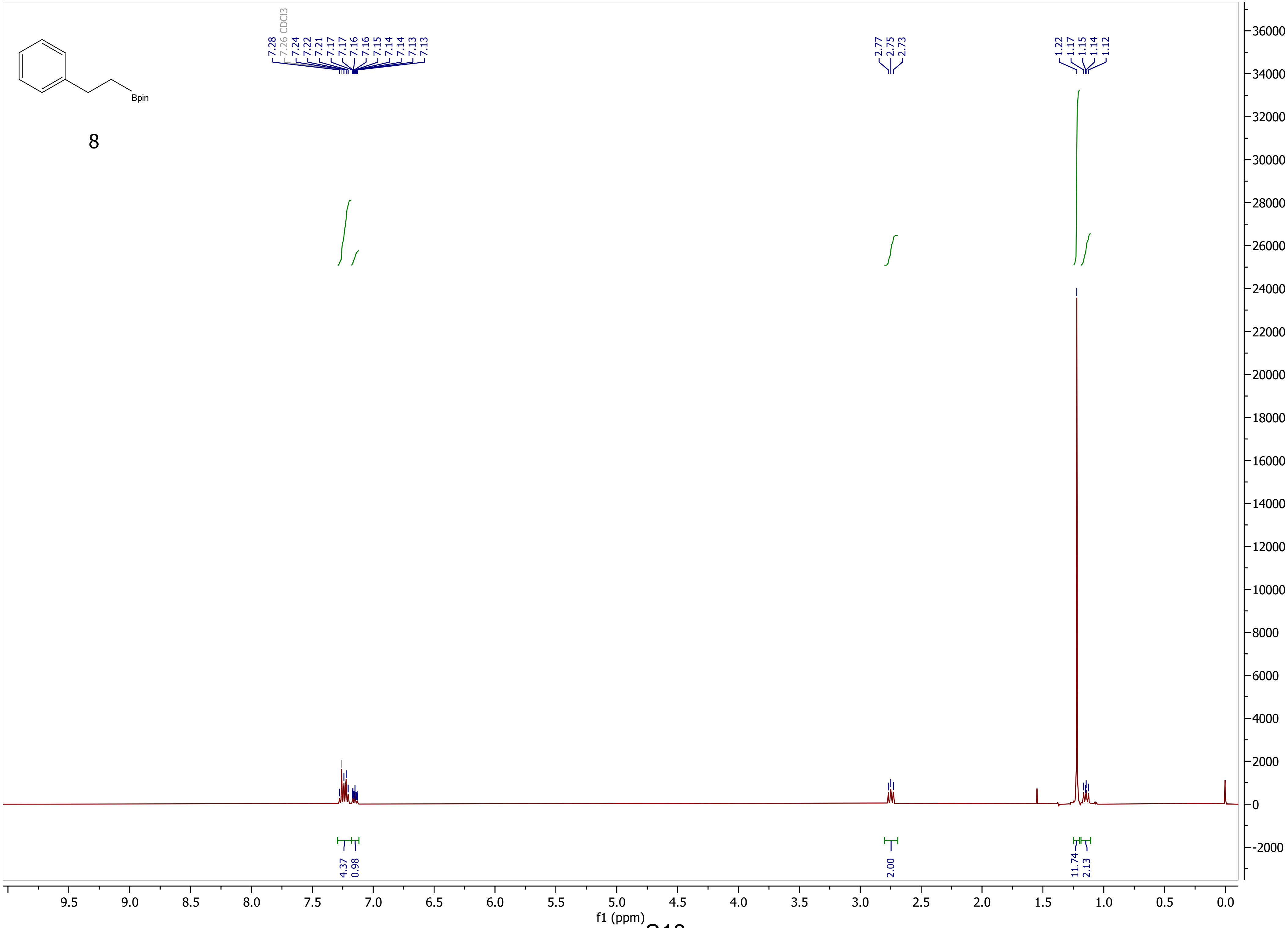

Bpin
8
7.28
7.26 CDCl3
7.24
7.22
7.21
7.17
7.17
7.16
7.16
7.15
7.14
7.14
7.13
7.13
2.77
2.75
2.73
1.22
1.17
1.15
1.14
1.12
4.37
0.98
2.00
11.74
2.13
f1 (ppm)

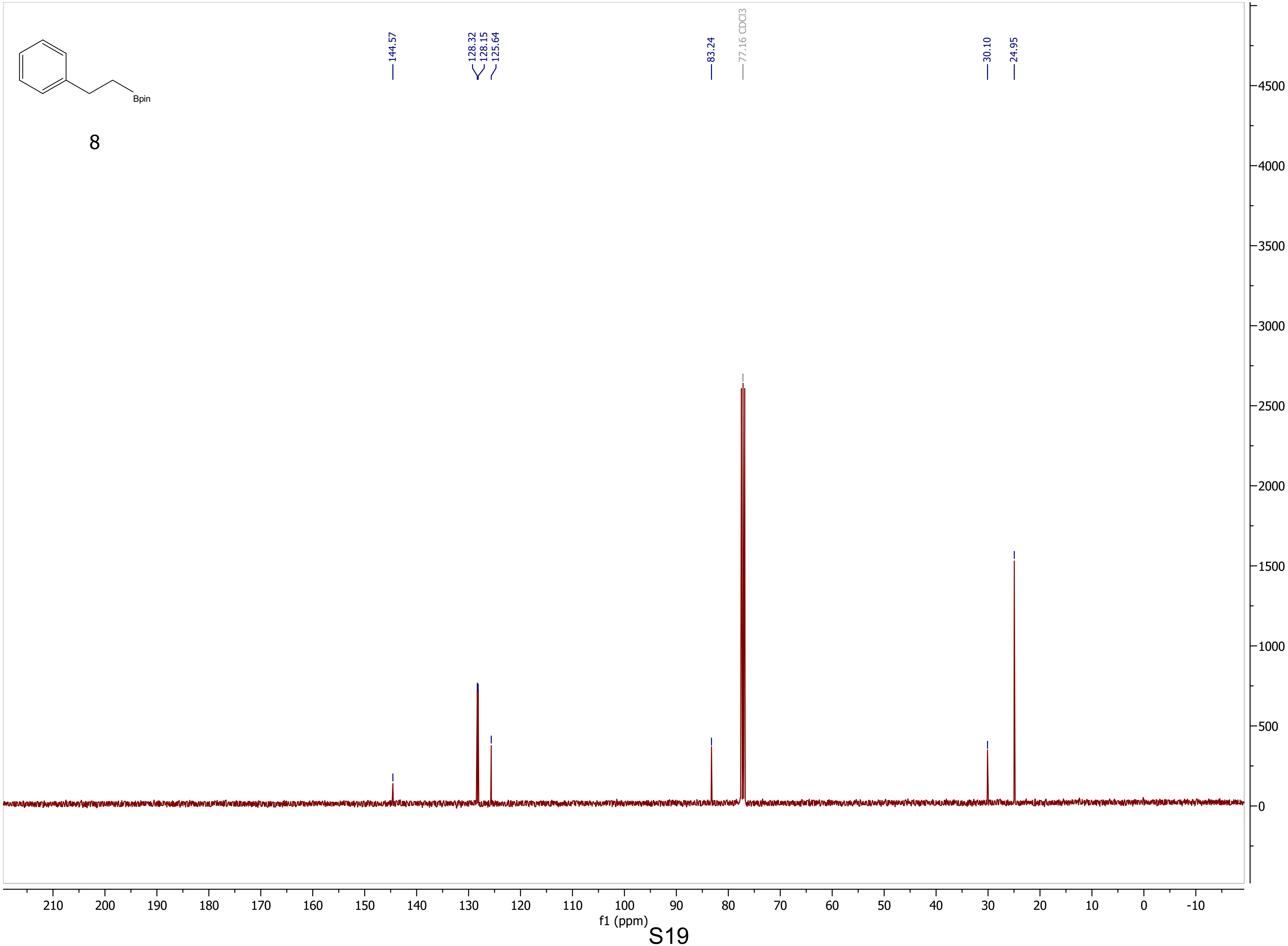

Bpin
8
144.57
128.32
128.15
125.64
83.24
77.16 CDCl3
30.10
24.95
f1 (ppm)

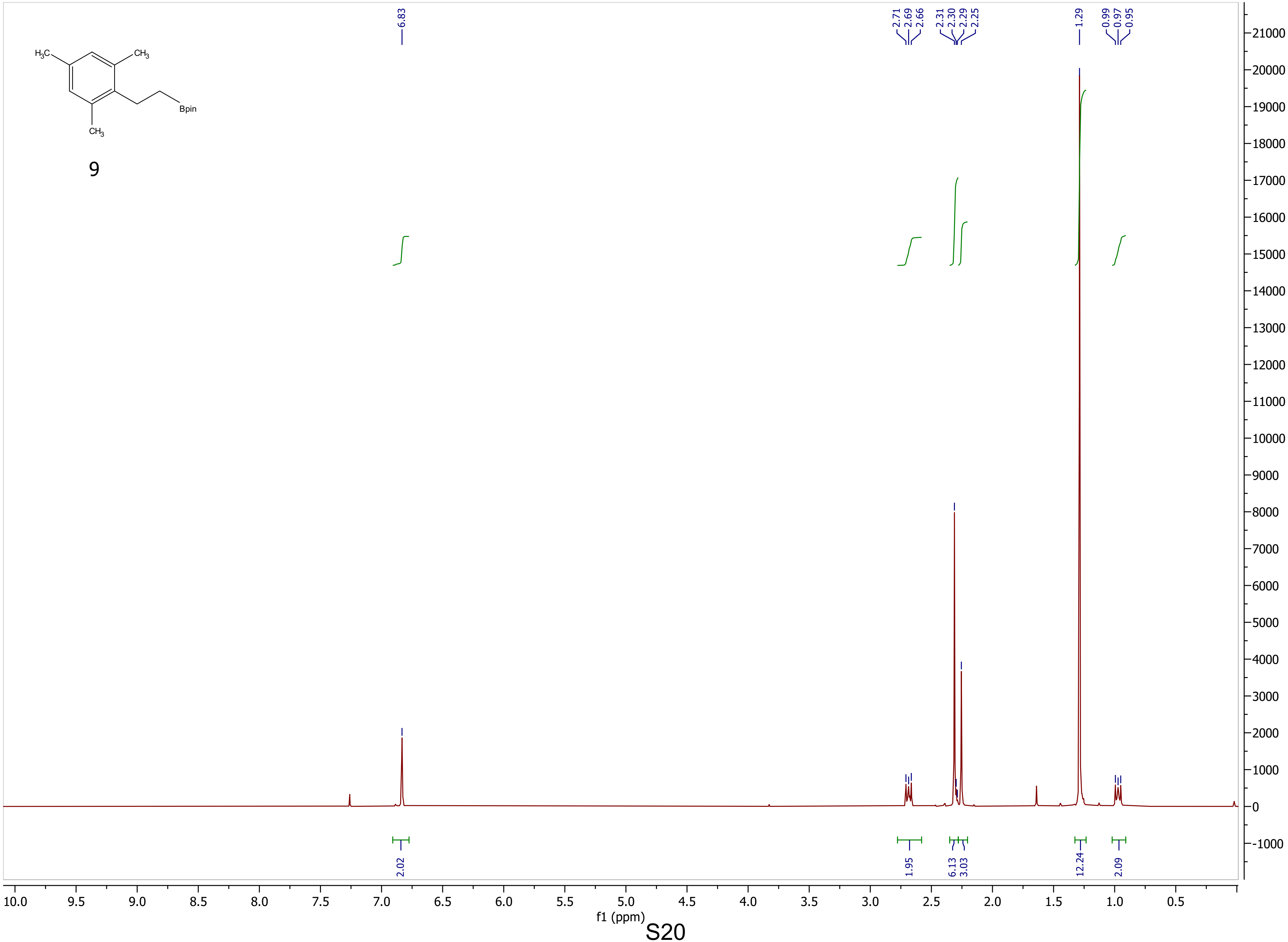

H3C
CH3
Bpin
CH3
9
6.83
2.71
2.69
2.66
2.31
2.30
2.29
2.25
1.29
0.99
0.97
0.95
2.02
1.95
6.13
3.03
12.24
2.09
f1 (ppm)

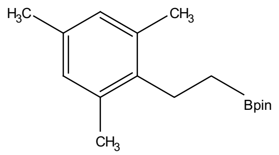
H3C
CH3
Bpin
CH3

9

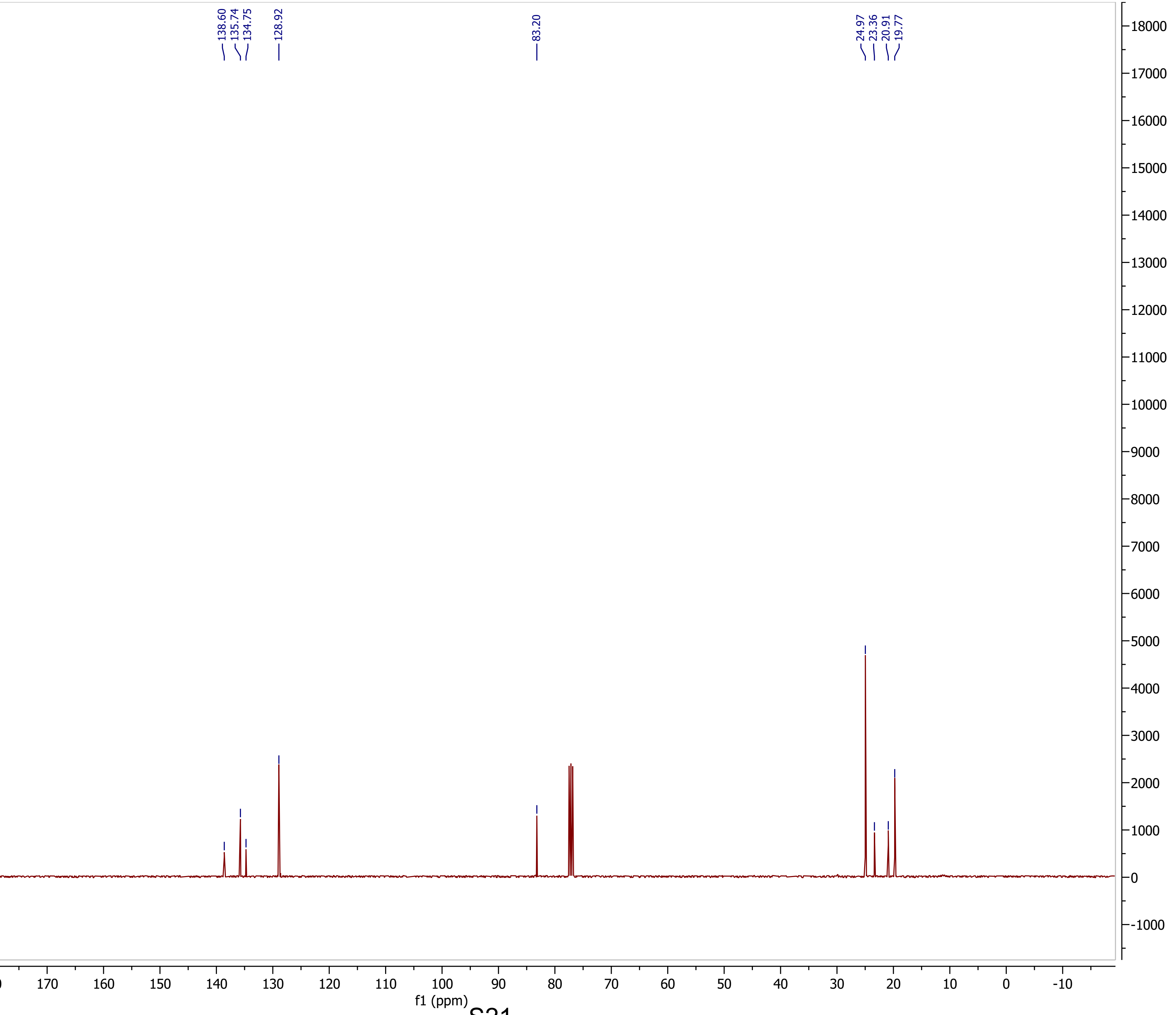
138.60
135.74
134.75
128.92
83.20
24.97
23.36
20.91
19.77
f1 (ppm)

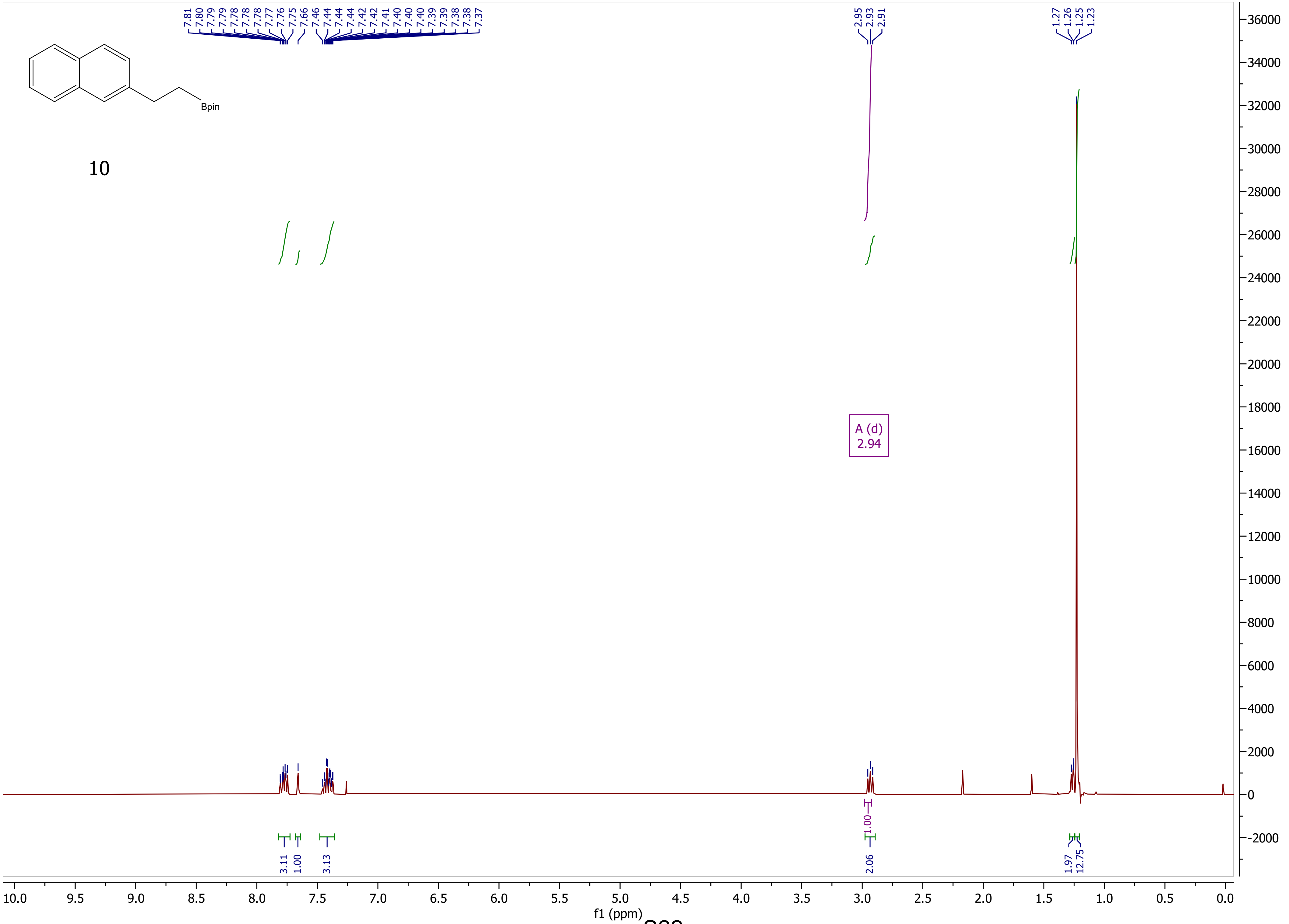
Bpin
10
7.81 7.80 7.79 7.79 7.78 7.78 7.78 7.77 7.76 7.75 7.66 7.46 7.44 7.44 7.44 7.42 7.42 7.41 7.40 7.40 7.39 7.39 7.38 7.38 7.37
2.95 2.93 2.91
1.27 1.26 1.25 1.23
A (d)
2.94
3.11 1.00 3.13 1.00 2.06 1.97 12.75
f1 (ppm)

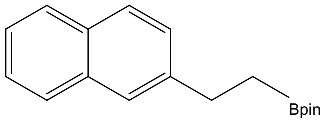
Bpin

10

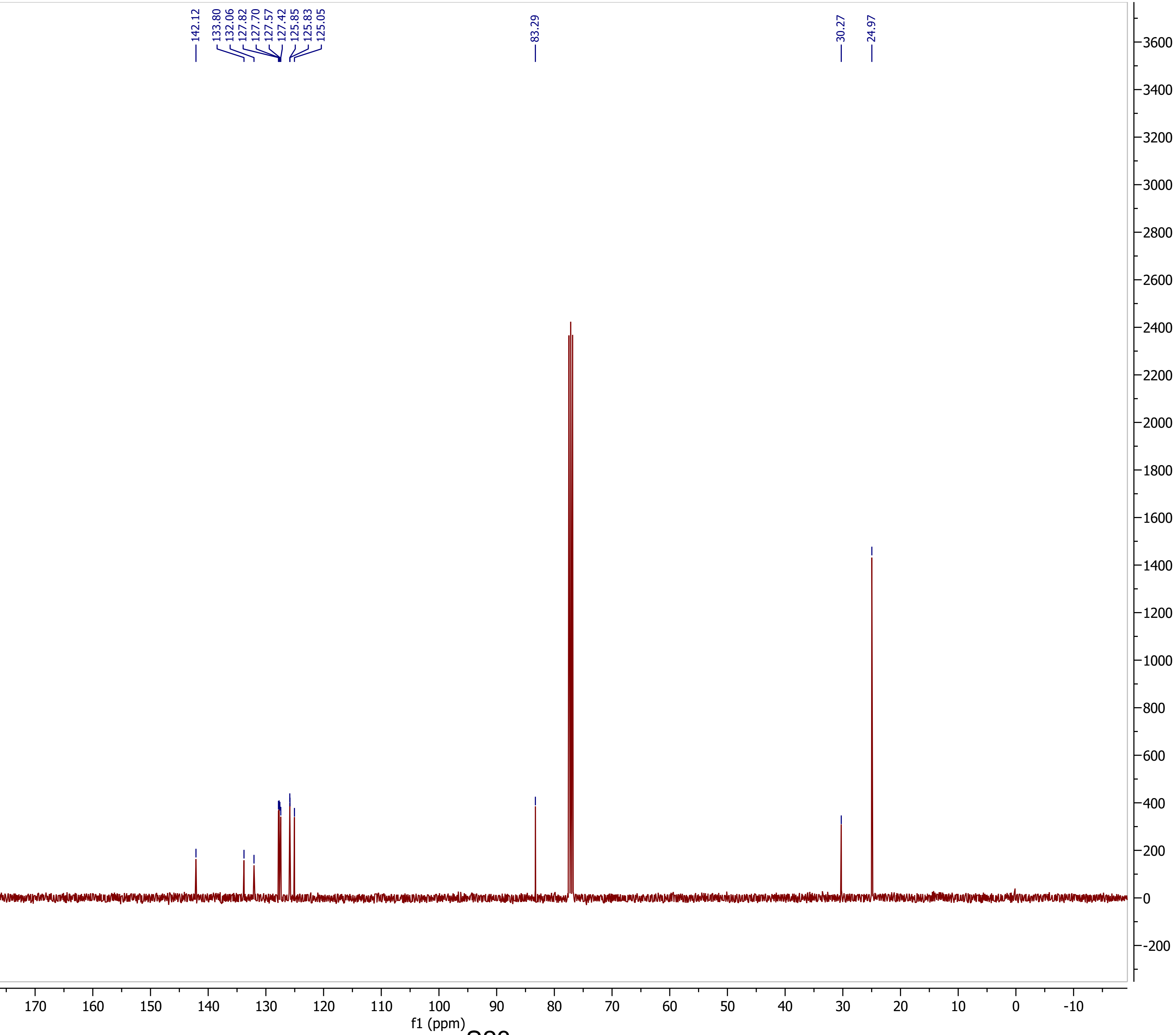
142.12
133.80
132.06
127.82
127.70
127.57
127.42
125.85
125.83
125.05
83.29
30.27
24.97
f1 (ppm)

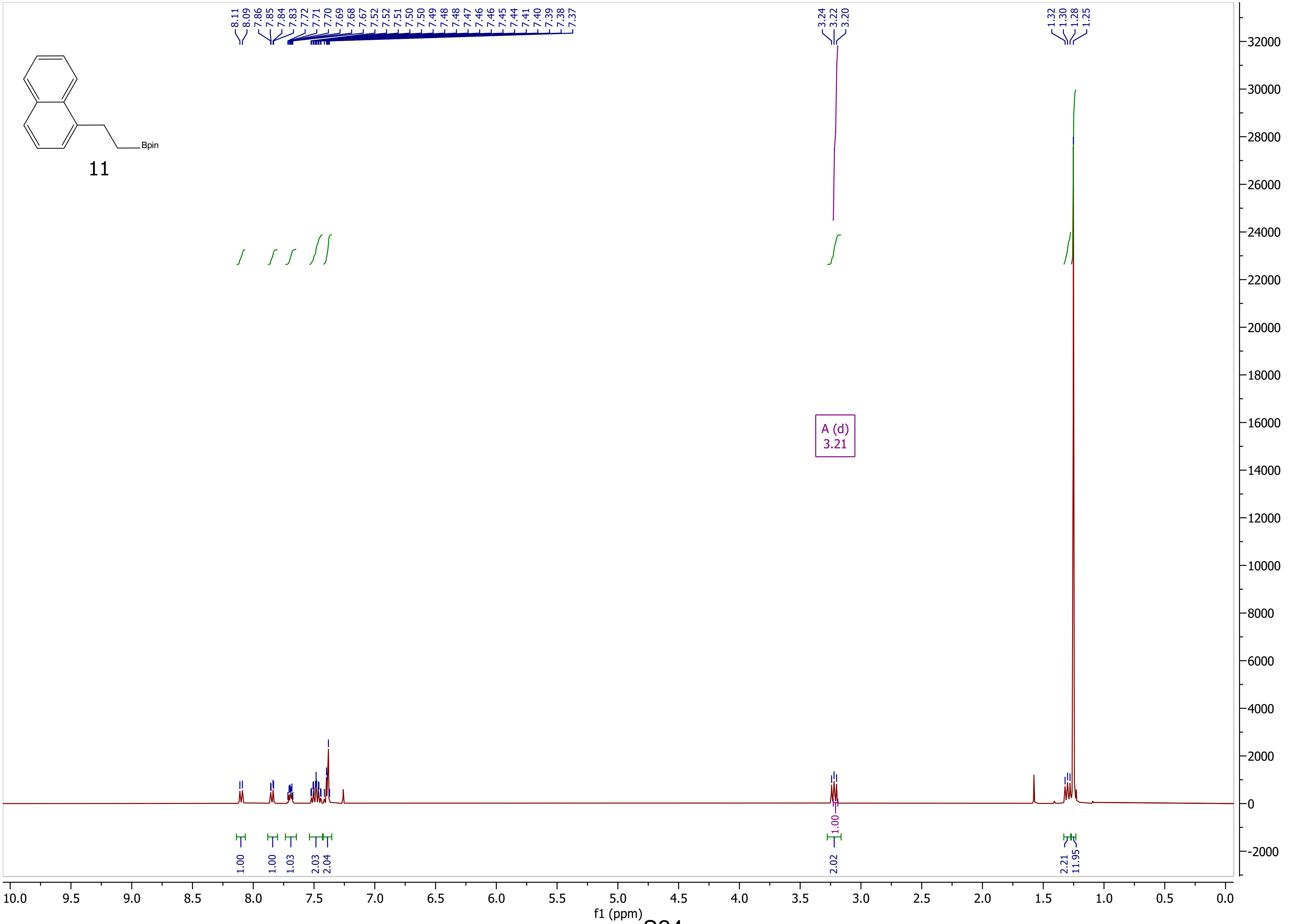
Bpin
11
8.11
8.09
7.86
7.85
7.84
7.83
7.72
7.71
7.70
7.69
7.68
7.67
7.52
7.52
7.51
7.50
7.50
7.49
7.48
7.48
7.47
7.46
7.46
7.45
7.44
7.41
7.40
7.39
7.38
7.37
3.24
3.22
3.20
1.32
1.30
1.28
1.25
A (d)
3.21
1.00
1.00
1.00
1.03
2.03
2.04
2.02
2.21
11.95
f1 (ppm)

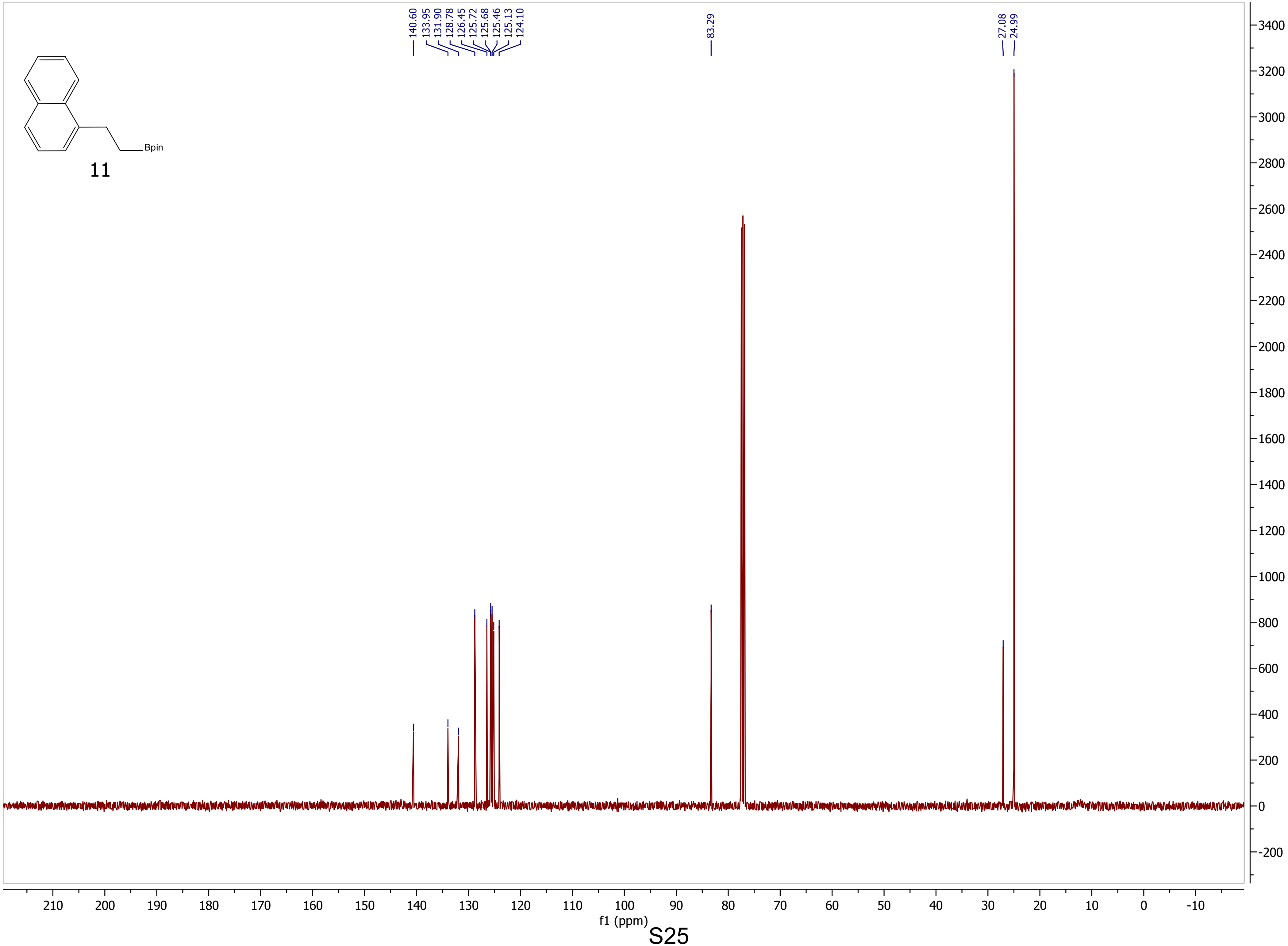
Bpin
11
140.60
133.95
131.90
128.78
126.45
125.72
125.68
125.46
125.13
124.10
83.29
27.08
24.99
3400
3200
3000
2800
2600
2400
2200
2000
1800
1600
1400
1200
1000
800
600
400
200
0
-200
210 200 190 180 170 160 150 140 130 120 110 100 90 80 70 60 50 40 30 20 10 0 -10
f1 (ppm)

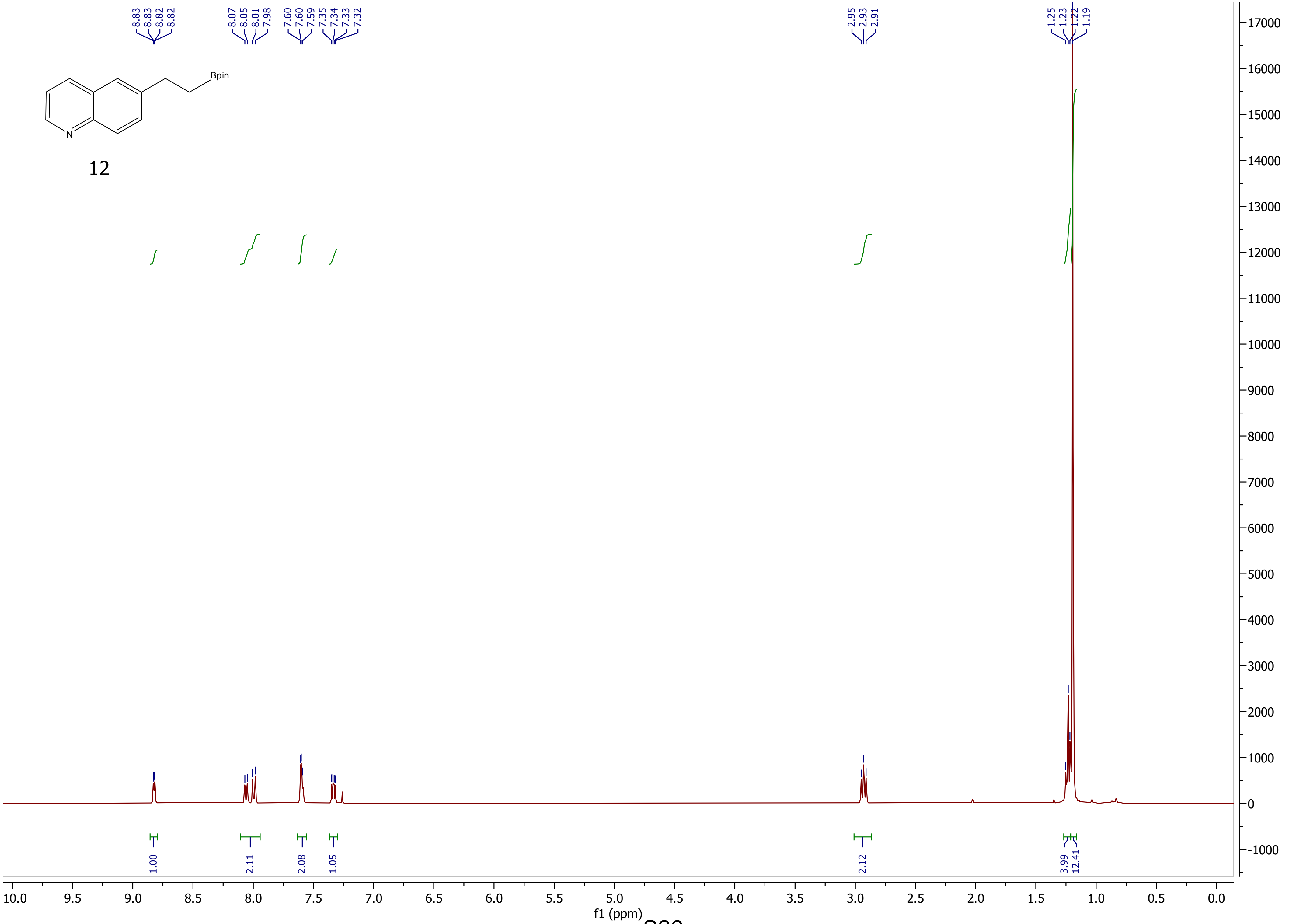
Bpin
N
12
8.83
8.83
8.82
8.82
8.07
8.05
8.01
7.98
7.60
7.60
7.59
7.35
7.34
7.33
7.32
2.95
2.93
2.91
1.25
1.23
1.22
1.19
1.00
2.11
2.08
1.05
2.12
3.99
12.41
f1 (ppm)

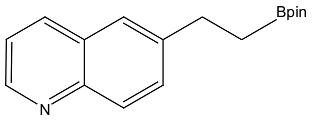
Bpin
N

12

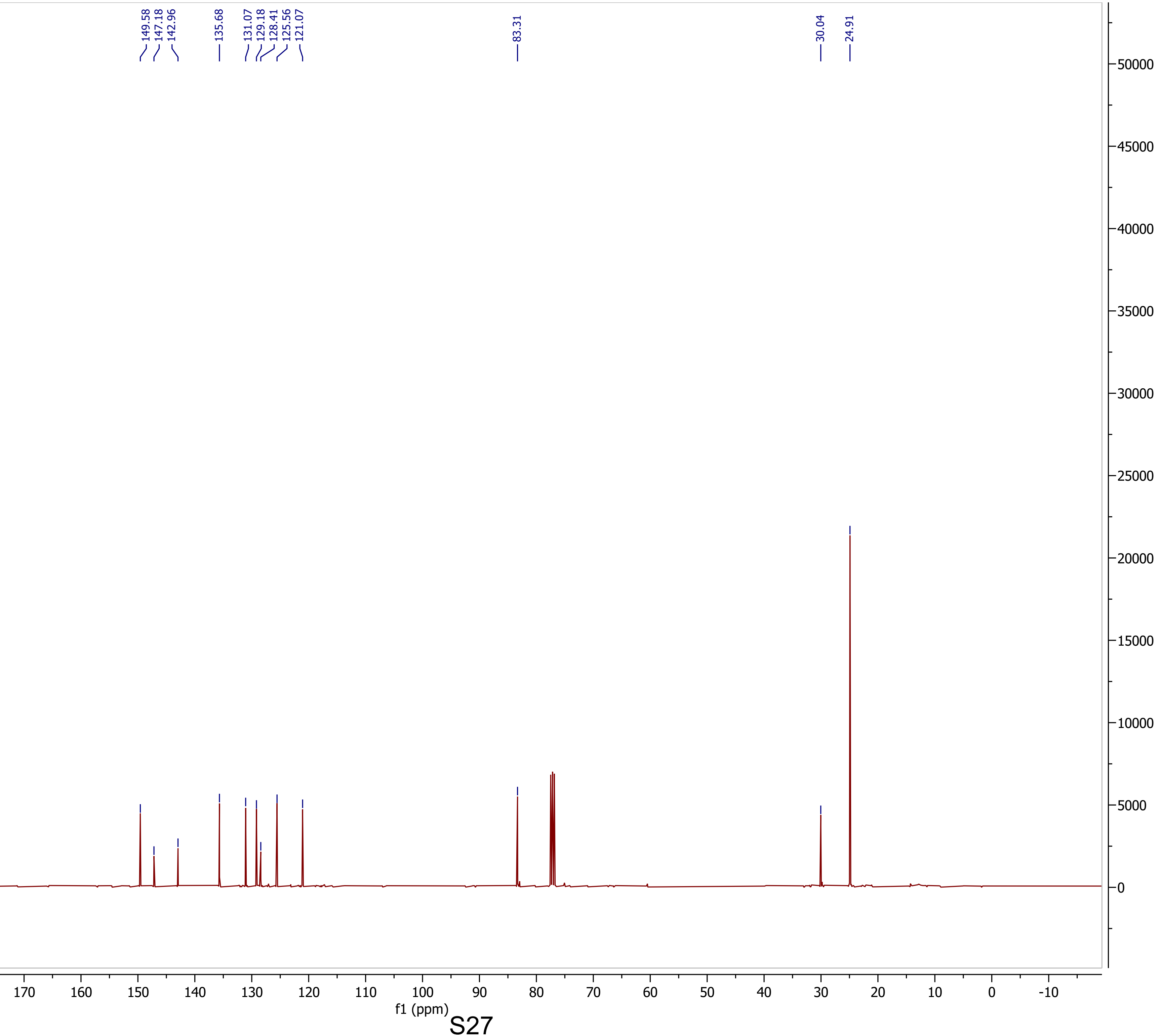
149.58
147.18
142.96
135.68
131.07
129.18
128.41
125.56
121.07
83.31
30.04
24.91
50000
45000
40000
35000
30000
25000
20000
15000
10000
5000
0
210
200
190
180
170
160
150
140
130
120
110
100
90
80
70
60
50
40
30
20
10
0
-10
f1 (ppm)

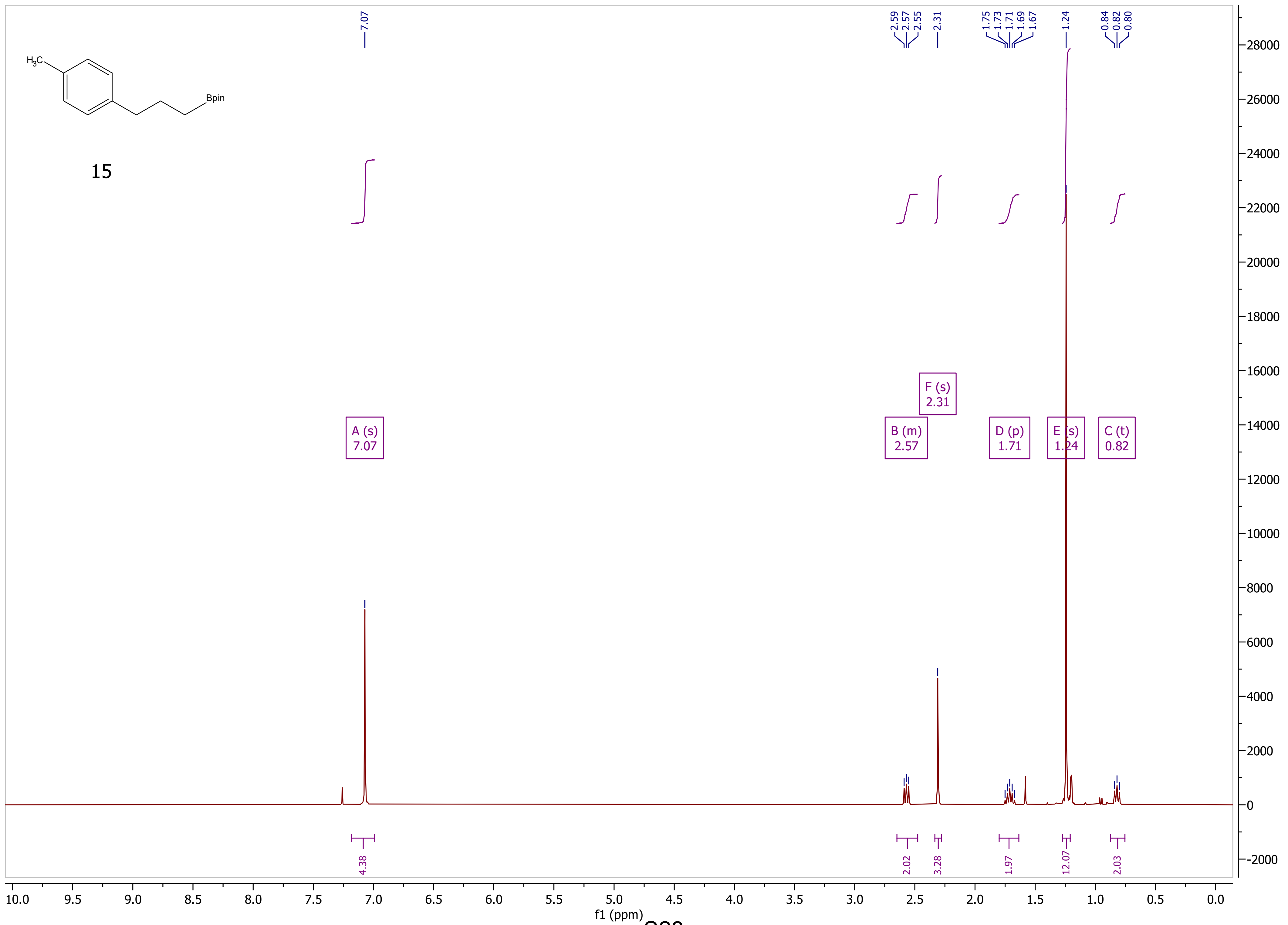

H3C
Bpin
15
7.07
2.59
2.57
2.55
2.31
1.75
1.73
1.71
1.69
1.67
1.24
0.84
0.82
0.80
A (s)
7.07
B (m)
2.57
F (s)
2.31
D (p)
1.71
E (s)
1.24
C (t)
0.82
4.38
2.02
3.28
1.97
12.07
2.03
f1 (ppm)

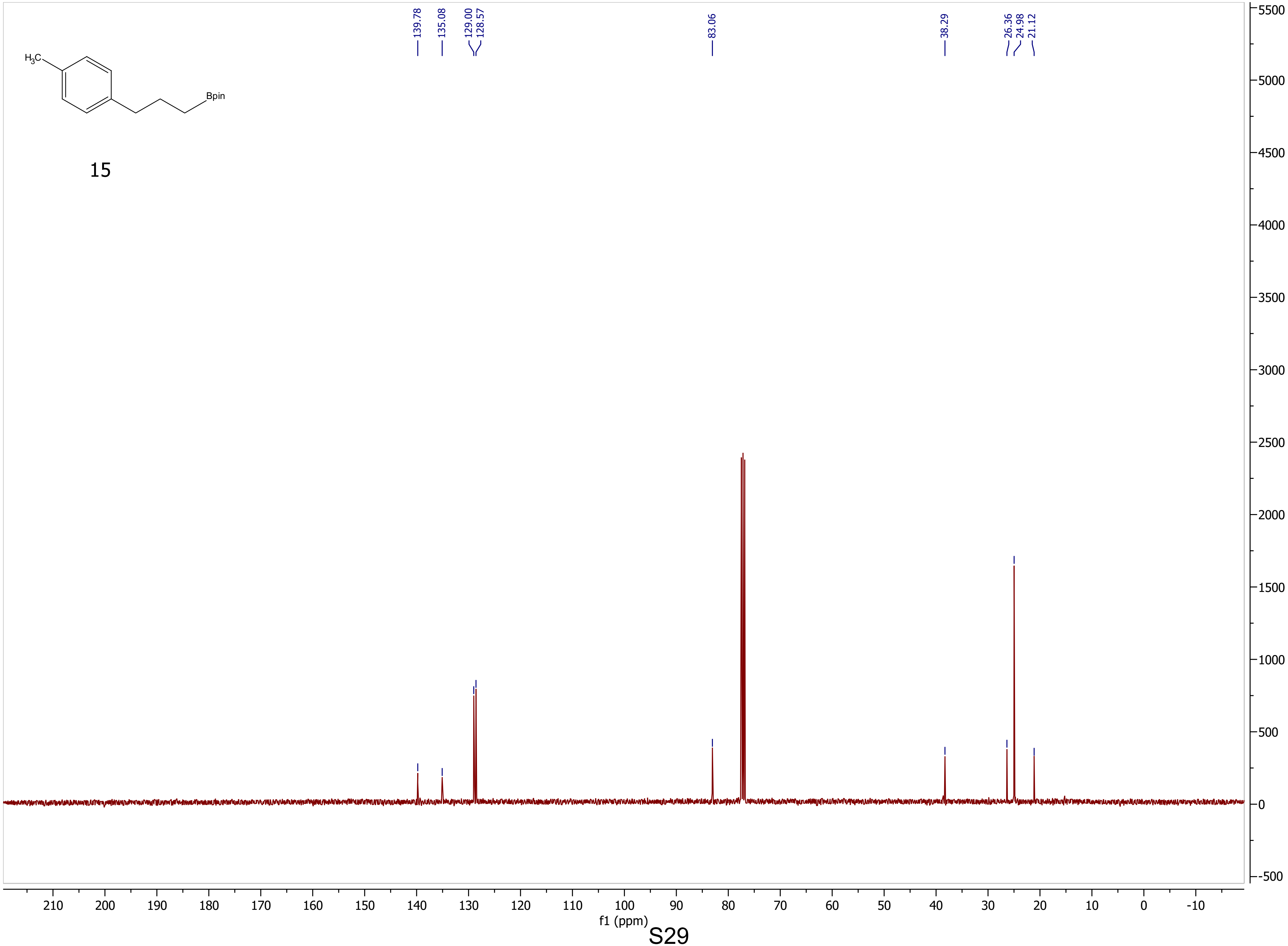

15

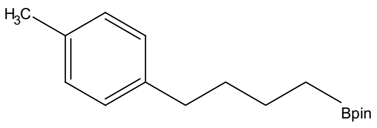
H3C
Bpin

16

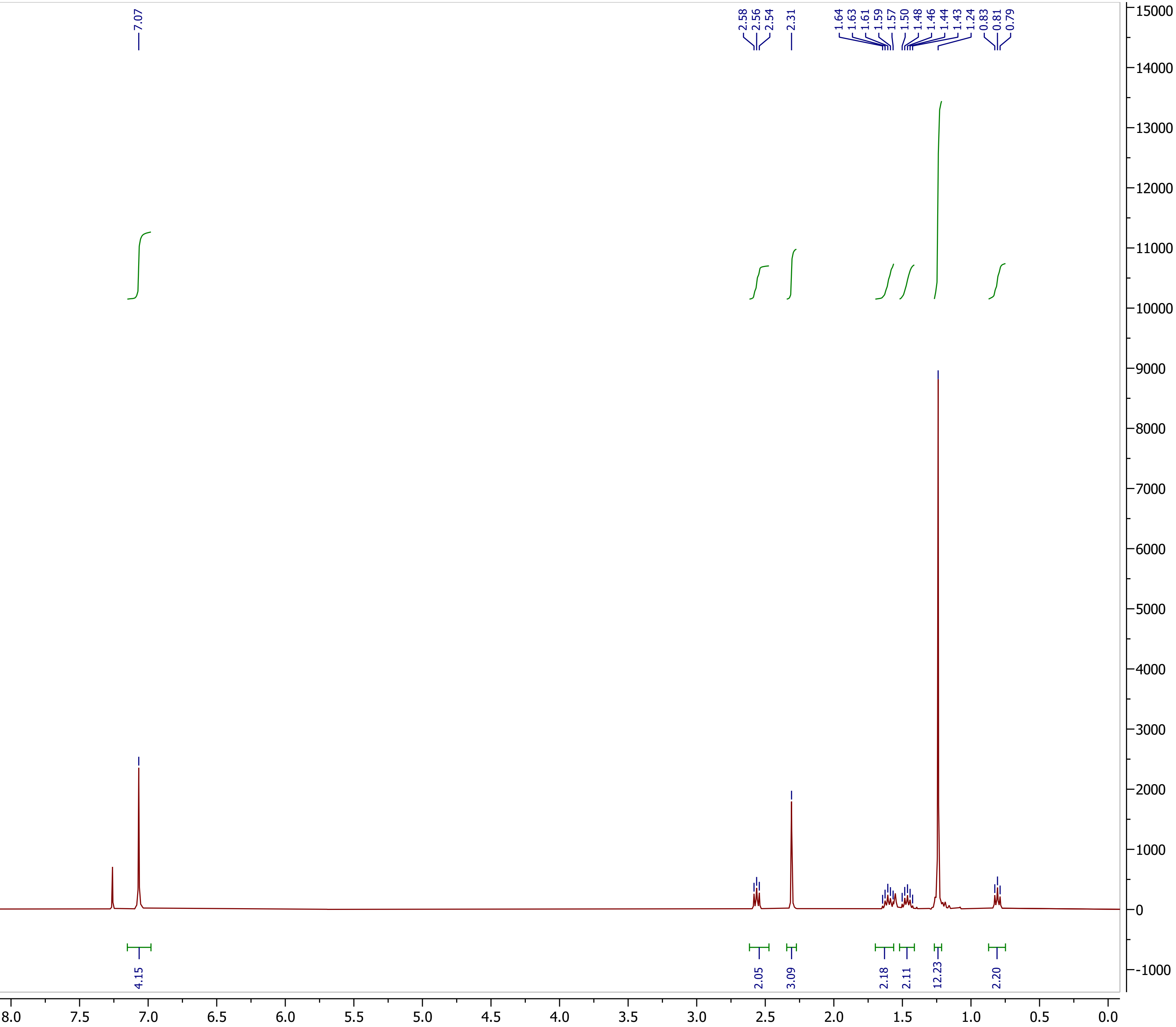
7.07
2.58
2.56
2.54
2.31
1.64
1.63
1.61
1.59
1.57
1.50
1.48
1.46
1.44
1.43
1.24
0.83
0.81
0.79
4.15
2.05
3.09
2.18
2.11
12.23
2.20
15000
14000
13000
12000
11000
10000
9000
8000
7000
6000
5000
4000
3000
2000
1000
0
-1000
10.0 9.5 9.0 8.5 8.0 7.5 7.0 6.5 6.0 5.5 5.0 4.5 4.0 3.5 3.0 2.5 2.0 1.5 1.0 0.5 0.0
f1 (ppm)

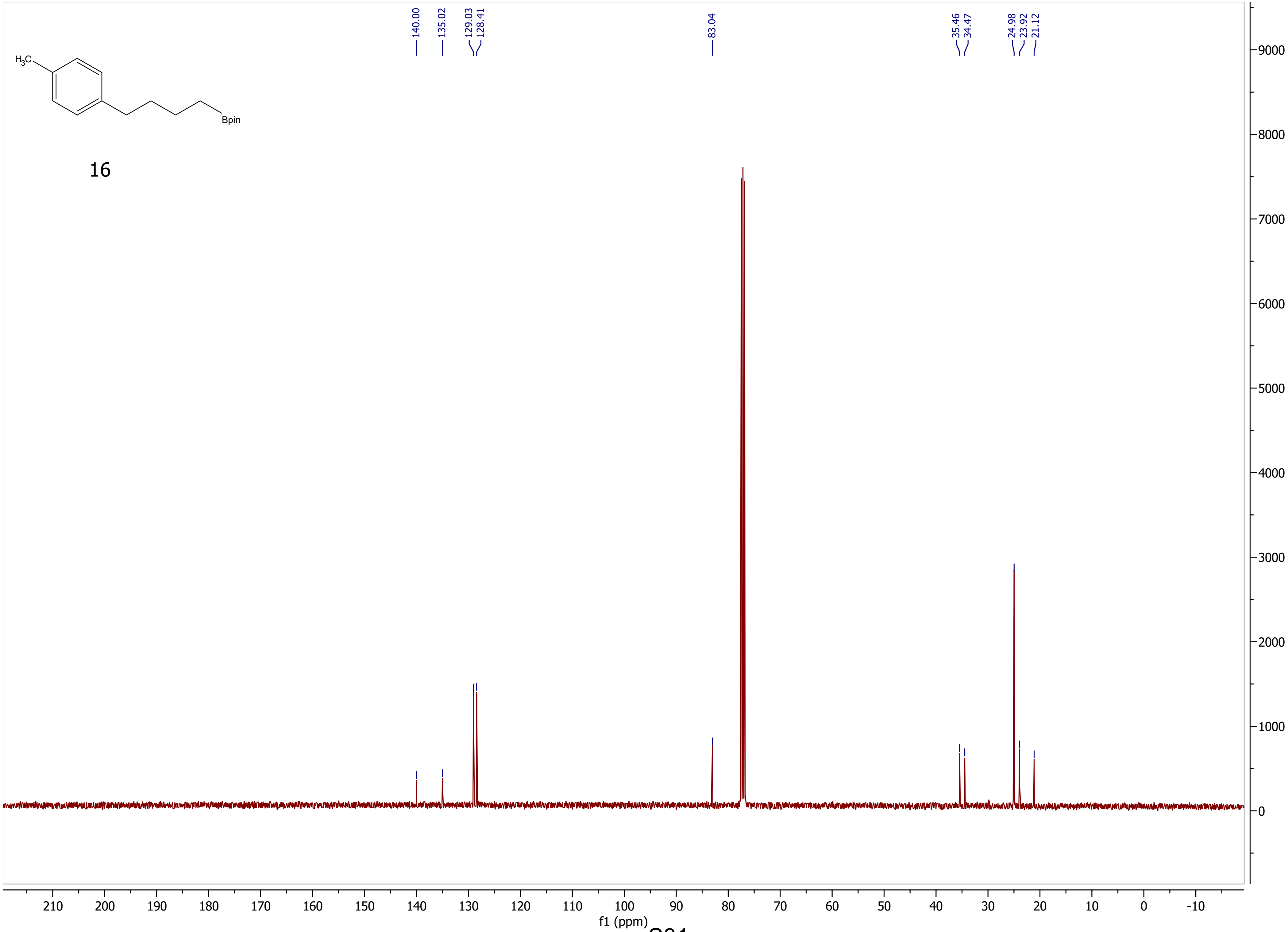

H3C
Bpin
16
140.00
135.02
129.03
128.41
83.04
35.46
34.47
24.98
23.92
21.12
f1 (ppm)
